# Encapsulation of reactive species within metal–organic cages

**DOI:** 10.1039/d5sc02081f

**Published:** 2025-07-22

**Authors:** Soumalya Bhattacharyya, Martin R. Black, Ben S. Pilgrim

**Affiliations:** a School of Chemistry, University of Nottingham University Park Nottingham NG7 2RD UK ben.pilgrim@nottingham.ac.uk

## Abstract

Reactivity under confinement often differs greatly from reactivity in the bulk. Metal–organic cages (MOCs) are a class of discrete, solution-processable container molecules encompassing well-defined nanospaces, which can be rapidly constructed in modular fashion *via* self-assembly. Supramolecular chemists have created an extensive library of MOCs and demonstrated their ability to serve as molecular flasks, with cavities tailored to bind guests of interest. In this review, we cover selected examples of the encapsulation and relative stabilisation of reactive species within MOCs, from early reports to the most recent developments. Most reactive species are not inherently unstable; but they persist only as long as they do not encounter a partner with whom they can react. MOCs can prevent or reduce the rate of this deleterious reactivity through acting as a shield and providing a physical barrier between an encapsulated reactive guest and other system components regularly encountered in the bulk environment, including air, water, solvent, light, another molecule of itself, or a co-reactant. Thus, MOCs can extend the lifetime of these short-lived reactive species enhancing their study, or allowing for different reactivity to be explored. Examples have been segregated based on the nature of stabilisation (*i.e.*, with what partner a reaction has been prevented). We believe this analysis will help provide more nuanced understanding of what types of highly reactive species can be tolerated within a dynamic MOC system to enable MOCs to find use in a wider variety of real-world applications.

## Abstract 2

Reactivity in confinement differs from bulk. Metal–organic cages (MOCs) can confine reactive species, shielding them from unwanted reactions and extending their lifetimes. Here we explore how MOCs stabilise such species and control their reactivity.

## Graphical abstract

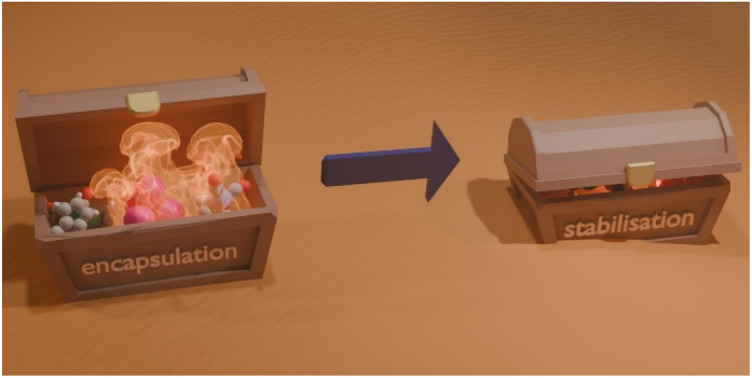

## Introduction

Metal–organic cages (MOCs) are “nanoboxes” – containers on the molecular scale.^1^ MOCs act as hosts and can encapsulate a range of guest molecules within their well-defined cavities,^2^ which range from volumes of ∼100 Å^3^ to ∼100 000 Å^3^. MOCs can often be constructed quantitively from their components in one pot under relatively mild conditions. This contrasts with some other porous molecules such as organic macrocycles, the synthesis of which can be laborious, harsh, and low yielding. MOCs are held together by coordination interactions between their constituent parts: metal ions (M) and organic linkers/ligands (L).^3^ They are formed under the reversible process of self-assembly, *i.e.* they are typically the most thermodynamically stable state of the system. However, as coordination bond formation is reversible, the geometries of the component parts must be precisely defined to favour construction of a singular product. Like a child's construction set, chemists typically work with pieces of set angles and shapes. Symmetry is also incredibly helpful and allows the same piece to be used multiple times within a structure reducing selectivity issues of which pieces join.

**Fig. 1:**
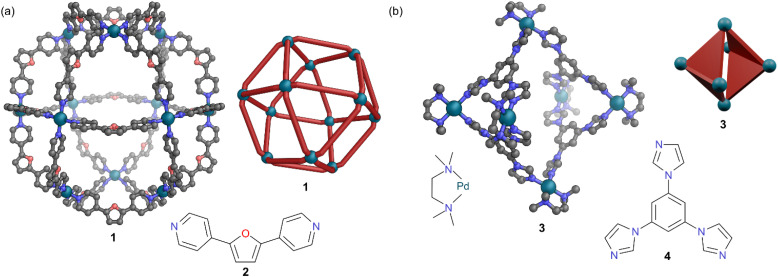
Representation of the SCXRD structures of (a) cage 1, and (b) cage 3, alongside cartoon representations and their respective ligands 2 and 4. Disorder, solvent, hydrogen atoms, and counter anions have been omitted for clarity. Colour: C = grey, N = blue, O = red, Pd = turquoise.

A wide range of MOCs have been constructed following these principles, with many of the common polyhedral shapes (tetrahedra, cubes, octahedra, prisms *etc.*) having been accessed.^4^ The polyhedra often have metal ions at the vertices and ligands along the edges. The precise shape which forms depends upon the ligand coordination vectors (angle between the bonds linking the ligand donor atoms to the metals) and how these fit with the coordination or chelate plane of different metal ions. This is termed the symmetry interaction model.^5^ For example, cuboctahedral MOC 1 with square planar Pd(ii) ions at the vertices, requires a bend angle of 120° in ligand 2 to match the angle between metal coordination planes at adjacent vertices (Fig. 1a). MOCs can also be constructed with metal ions at the vertices and two-dimensional ligand panels covering some or all the faces. An example of this molecular panelling approach sees four of the eight triangular faces of octahedral MOC 3 covered with triangular ligand 4, with *N*,*N*,*N*′,*N*′-tetramethylethylenediamine (TMEDA) capping the remaining two coordination sites on each Pd(ii) ion (Fig. 1b).^6^ Other notable approaches include the directional bonding model, which have been discussed in detail in other reviews.^7^

A key feature of MOCs is their highly modular nature; their components can be easily tailored to impart properties onto the hollow cavity suitable for guest binding. Guest-cavity interactions are typically non-covalent in nature. Designing a cavity to enhance guest binding includes both modifying bulk cavity properties such as hydrophobicity or electrostatic potential (*i.e.*, positively charged or negatively charged MOC), and modifying more specific directional attractions between the walls of the cavity and guests such as hydrogen bonding,^8^ π–π stacking,^9^ or halogen bonding interactions.^10^

**Fig. 2:**
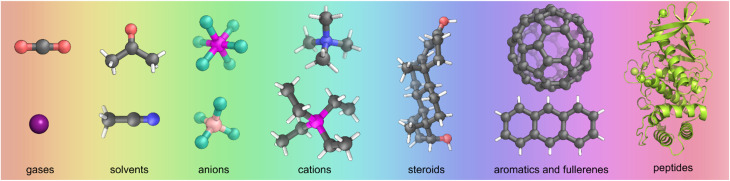
A selection of common unreactive guest molecules often bound in MOCs, including the gases xenon and carbon dioxide, solvents acetone and acetonitrile, anions hexafluorophosphate and tetrafluoroborate, cations tetramethylammonium and tetraethylphosphonium, steroid epitestosterone, buckminsterfullerene and anthracene, and peptide thermolysin. Colour: C = grey, N = blue, O = red, H = white, F = aquamarine, P = magenta, B = pink, Xe = dark purple. Thermolysin is shown as a cartoon representation.

While guests successfully encapsulated vary greatly in size from single atoms^11^ to small proteins,^12^ in terms of functionality certain classes of guests have been highly favoured. The construction of MOCs requires reversible metal–ligand bond formation, and this dynamicity often persists in the final structure. Guests chosen to probe binding behaviour have thus typically been unreactive, to not disrupt these metal–organic linkages which can be fragile. Guests are also often poorly soluble in the solvent in which the MOC is dissolved to provide an extra driving force for encapsulation. Some popular guest classes for MOCs that fall into these unreactive and poorly soluble categories include aromatic hydrocarbons, fullerenes, and steroids (Fig. 2). Unreactive gases, such as Xe,^11^ SF_6_,^13^ and CO_2_ ^14^ have been bound without reacting. Whereas with more reactive gases such as SO_2_,^15^ studies have focussed on the gas reacting once bound. In the absence of more strongly competing guests, MOC cavities in solution are filled with solvent; residual solvent is often found in the cavity in single crystal X-ray diffraction (SCXRD) structures. Most MOCs carry an overall charge and as materials are themselves salts with corresponding counterions. These counterions are typically chosen to be non-coordinating to not compete with metal–ligand interactions, and one or more of these counter cation or anions can often be found encapsulated as a guest. Non-coordinating ions are often added to investigate binding behaviour or promote crystallisation. Other common classes of unreactive guests include dyes and biomolecules (peptides/small proteins). Whilst certain useful problems can be tackled through the binding of these relatively unreactive guests, for example the purification of hydrocarbon feedstocks,^16^ many other important potential applications, including catalysis, drug delivery, sensing, and artificial light-harvesting require MOCs to bind more highly reactive species in more challenging environments.

The microenvironments generated in a confined cavity are often completely different from conventional solid, liquid, or gaseous phases, altering the behaviour of bound guests. When a reactive guest is bound, the MOC walls provide a physical barrier to encounters with bulk solvent molecules or other reactive components in the system, often significantly suppressing reactivity. We draw a contrast here with the far more widespread use of MOCs for catalysis, where the MOCs are designed to increase reactivity upon encapsulation (enzyme active site-esque) rather than decrease reactivity. This has been reviewed extensively elsewhere and will not be discussed here.^17^ Whilst the benefits of increasing reactivity upon encapsulation are obvious, the benefits of decreasing reactivity are also profound. Such prevention from onwards reaction can substantially increase the lifetime of reactive species, enabling more detailed study of the nature of these (sometime elusive) intermediates. The information obtained can shed new light on reaction mechanisms. Suppressing an unwanted reactivity pathway can also allow an alternative pathway, normally too uncompetitive to be observed, to instead predominate, providing unusual reaction selectivity. This review will seek to summarise where the encapsulation and relative stabilisation of reactive species has led to interesting outcomes.

The stabilisation of short-lived/reactive species in supramolecular container molecules is a broad subject and was reviewed in 2016 by Ballester and co-workers.^18^ In this review we focus solely on MOCs, as many new examples have been reported since 2016. In addition, the dynamic nature of the coordination linkages within MOCs makes the encapsulation of reactive species within these porous materials a unique challenge, as these reactive encapsulated species must not interfere with these coordination interactions.

Before considering individual examples, we first briefly consider the robustness/fragility of MOCs to various reagents and conditions, as knowledge of this is crucial to plan where encapsulation of reactive species can be successful. It is important to stress here that the reactive species are not inherently unstable – they are stable species existing in their own energy minima (even if this is shallow). In isolation they could exist indefinitely; they decompose through interaction with another component of the system such as solvent, oxygen, or light. Upon encapsulation within a suitably designed MOC, these unwanted interactions can be reduced.

We have divided the examples into five sections based on the nature of protection provided by the MOC: protection against reaction with (i) solvents; (ii) air; (iii) other chemicals; (iv) light; and (v) the case of MOCs altering functional group reactivity (typically through partial encapsulation, *i.e.*, promoting selectivity for reaction of a normally less reactive group by shutting off reactivity of a more reactive functional group). It should be noted however, that many MOCs protect their guest against several of these deleterious reactivity pathways simultaneously. The range of reactive guests bound is vast, including inorganic, organic, and organometallic species, that are anionic, cationic, and neutral.

## The stability of MOCs to reactive species

Most reactive species are not inherently unstable; they exist in an energy minimum but with a generally low energy pathway to react. If surmounting the local maxima to get out of the well is easy, then they react and form something else. In most cases discussed, the reactive species need to either find a co-reactant to participate in this low energy reaction pathway or receive extra energy from a photon of light; their decomposition is not a unimolecular process driven purely by thermal energy.

It is useful to think about common classes of reactivity at this point, as this is instructive in how this reactivity can be supressed, but also what characteristics any MOC would need to have to encapsulate a reactive species of this nature. A reactive guest bound in a MOC cavity often sits nearest to atoms on the ligand backbone. These backbones are typically constructed from aryl rings due to their rigidity, and fortunately this often results in relatively unreactive functionality (such as aryl C–H bonds) pointing into the cavity.

### Strong electrophiles/acids

**Fig. 3:**
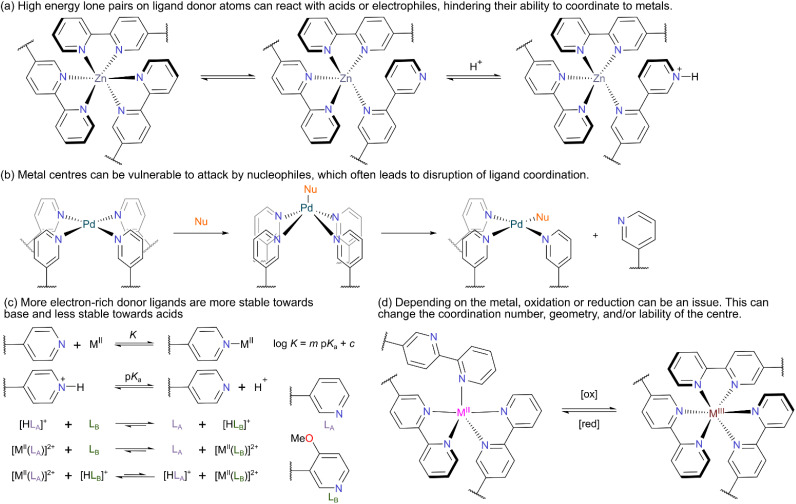
(a) Reversible ligation between a nucleophilic/basic donor atom and a metal centre; when deligated the donor atom can react with electrophiles. (b) Nucleophilic functionality can react with a metal centre, possibly leading to ligand displacement and degradation. (c) More electron-rich donor ligands form stronger interactions both with metal centres and protons. This makes more electron-rich ligands more stable towards base and less stable towards acid. (d) Redox agents can oxidise/reduce metal centres, with changes in coordination number or geometry leading to degradation.

Many reactive species are strongly electrophilic; they have empty non-bonding/antibonding orbitals that lie low in energy. Examples include acids (both Brønsted and Lewis), cations, and metal complexes with vacant sites. These species react with nucleophilic species with high energy pairs of electrons. In a MOC, the most nucleophilic components are typically the donor atoms of the constituent ligands. Whilst these are free during MOC formation, once formed they are tied up through coordination to the metal, reducing their nucleophilicity. MOCs exhibit a wide range of lability. Some are highly dynamic in solution, and when dissociation occurs, the lone pairs of the free ligands will likely make these MOCs unsuitable for encapsulation of reactive electrophiles such as H^+^ (Fig. 3a). Other MOCs are substitutionally inert, with any nucleophilic ligand donor groups strongly tied up once formed. These more inert MOCs are thus ideal candidates for binding reactive electrophiles. Where functional groups themselves have well known windows of pH stability (*e.g.*, imines), then MOCs constructed from such moieties often inherit this property, with imine-based cages typically stable down to about pH = 4 in water.^19^ Resistance to acids can be engineered through tailoring the ligand basicity or adding additional pendant basic groups to the ligand that are not involved in metal coordination.^20^ The sensitivity of MOCs to acid can also be exploited, with the switching of photoacids able to control the assembly and disassembly process.^21^

### Strong nucleophiles/bases

Many reactive species are strongly nucleophilic or basic; they have at least one high energy pair of electrons which makes them reactive towards electrophilic partners. The labile nature of the metal–ligand interaction makes metallosupramolecular species vulnerable to decomposition in the presence of strongly nucleophilic reagents. Generally, the metal centre is more vulnerable than the ligand and attack of the nucleophile at the metal centre can lead to ligand displacement (Fig. 3b). If the nucleophile binds more strongly this can be irreversible and lead to MOC decomposition. Both associative substitution mechanisms, for example at square planar centres, and dissociative substitution mechanisms, for example at many octahedral centres, are possible depending on the nature of the metal. Many solvents, particularly the ones commonly used to solubilise MOCs (such as MeCN, DMSO, and H_2_O) have nucleophilic lone pairs. Bulky Brønsted bases that only react with protons as electrophiles can also cause issue, as the species they deprotonate often then become stronger nucleophiles themselves (for example H_2_O to OH^−^). Again, the more inert MOCs will be better candidates for binding this type of reactive species.

Sigel and co-workers reported a linear relationship between the logarithm of the association constant of metal-pyridine complexes and the p*K*_a_ value of the corresponding pyridinium ion (Fig. 3c).^22^ Hence, in a system of two ligands, L_A_ and L_B_, where L_B_ is more basic than L_A_, ligand L_B_ will preferentially form both the pyridinium ion and the metal-pyridine complex. This leads to an interesting reversal of selectivity. Without acid, the [M^II^(L_B_)]^2+^ complex forms selectively. However, with acid, L_B_ is preferentially protonated, and the [M^II^(L_A_)]^2+^ complex predominates. Severin and co-workers examined this in systems of Pd(ii)-pyridine MOCs.^23^ MOCs constructed from more basic ligands (*e.g.*, L_B_) were more tolerant to the presence of free pyridine, but less tolerant to the presence of trifluoroacetic acid (TFA). When the amount of Pd(ii) was restricted, this enabled a pH-dependent switching between which structure formed. Due to this relationship, many pyridine-based MOCs are not stable in the presence of more nucleophilic/basic pyridine derivatives such as 4-dimethylaminopyridine (DMAP). However, upon addition of acids such as TsOH, the DMAP can be protonated preferentially and cages can reform.^24^ Likewise, nucleophilic anions such as chloride can lead to decomposition, but upon addition of Ag(i), AgCl is precipitated and the cage can reform. Crowley and co-workers also investigated vulnerability of Pd-pyridine MOCs to biologically-relevant nucleophilic moieties and found both cysteine thiol residues and histidine imidazole residues to be problematic.^25^

### Redox

All MOCs have a potential window in which they are redox stable. Outside of this window they can be oxidised or reduced, with the metal centre often being the site where redox occurs (electrons are frequently added to or removed from partially filled d-orbitals). It is rare for a particular metal centre to be both easily reducible and oxidisable. For example, the oxidation of Cu(i) centres often happens rapidly in air, however these Cu(i) centres have considerably more resistance to reduction, indicating reactive guests which are reductants might be better paired with these systems. Some MOCs have a wide potential window of stability and can resist both oxidation and reduction. Examples include d^10^ metal ions such as Zn(ii) and some low-spin d^6^ systems such as Fe(ii). Redox at the metal centre can change the preferred coordination number and coordination geometry; it can also change the lability of the metal centre. In most cases, these changes lead to unwanted structural changes in the MOC and decomposition (Fig. 3d). However, in other cases, oxidation has been employed to lock more labile Co(ii) systems to more robust Co(iii) systems post-assembly without changing the overall structure.^26^

### Light

Whilst photons of sufficient energy can lead to excitation of electrons in metal centres (many cages are coloured due to metal-to-ligand or ligand-to-metal charge transfer transitions), this is not often problematic, and this energy can be dissipated without MOC decomposition. Ligand functionality shows a wide range of sensitivity to light and this is usually predictable. If the MOC needs to withstand irradiation, then the use of more reactive ligand motifs can be avoided.

## MOCs preventing reaction with solvents

### Protection from water

**Fig. 4:**
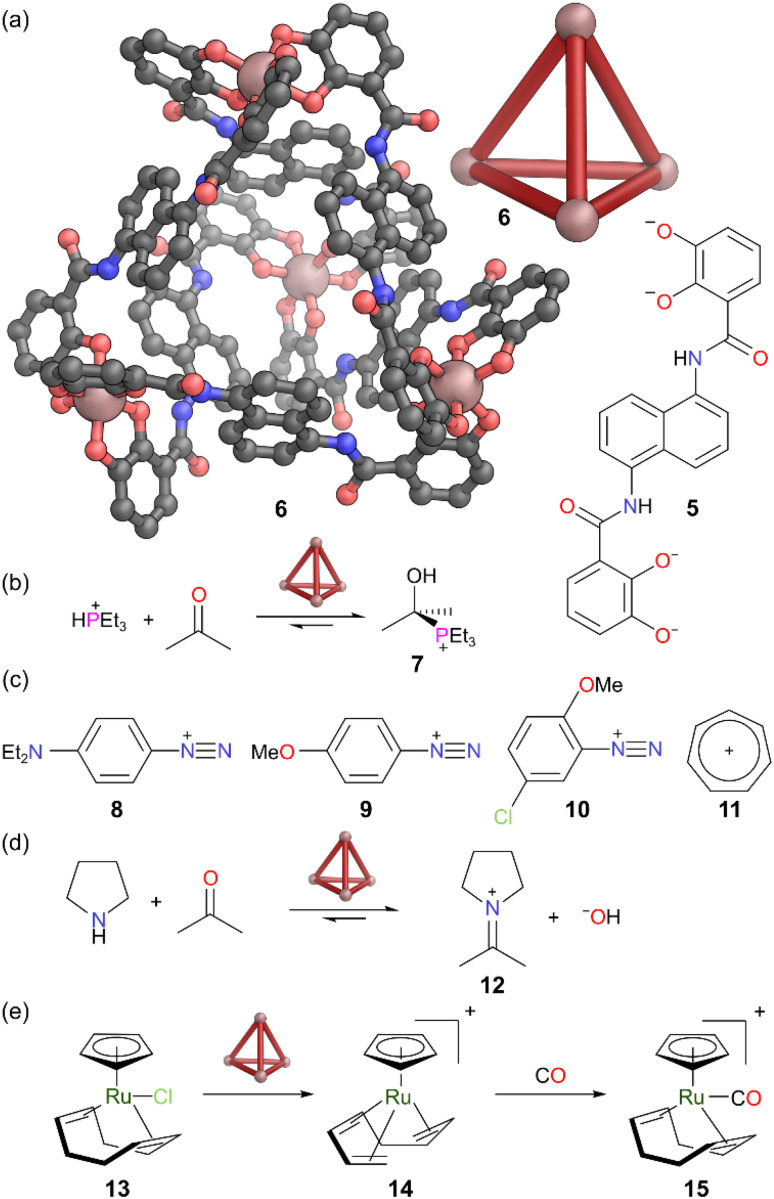
(a) Representation of the SCXRD structure of cage 6 constructed from ligand 5. (b) Formation of cationic adduct 7 is promoted by cage 6. (c) Cage 6 binds and stabilises diazonium cation 8 and tropylium cation 11 but does not bind diazonium cations 9 and 10. (d) Cage 6 stabilises iminium ions such as 12 which normally are transient in water. (e) Cage 6 promotes ionisation of Ru complex 13, to cationic complex 14 which binds. 14 reacts with CO to form 15 in the cavity. Disorder, solvent, hydrogen atoms, and counter cations have been omitted for clarity. Colour: C = grey, N = blue, O = red, Ga = burnt pink, P = magenta, Cl = light green, Ru = dark green.

Raymond and co-workers developed water soluble tetrahedral MOCs constructed from bidentate catecholamide ligands such as 5, with Ga(iii) or Fe(iii) as metal nodes (Fig. 4a). The ligands each possess a charge of 4−, giving a MOC of overall charge 12−. Anionic MOCs are much rarer than cationic MOCs and these systems have shown high proficiency for the encapsulation of a wide range of cationic guests in aqueous solution.^27^ Ga(iii)-based cage 6 has been explored for supramolecular encapsulation of reactive cationic species. The cationic adduct 7, formed from the reaction of triethylphosphine and acetone (Fig. 4b), had been previously synthesised and isolated in anhydrous conditions,^28^ but this had to be performed carefully as it readily decomposed back to initial reactants in the presence of water. However, [Ga_4_L_6_]^12−^ MOC 6 encapsulated adduct 7 in water forming the inclusion complex 7 ⊂ 6. The authors inferred that the encapsulated adduct 7 formed inside the MOC by diffusion in of protonated phosphine, HPEt_3_^+^, which reacted with encapsulated acetone molecules in the cage. This adduct then remained as a guest inside the cage after the use of acetone in the isolation process. The cage likely plays multiple roles here. Its anionic nature provides electrostatic stabilisation to the cationic adduct, lowering its relative energy compared to bulk solution. Whilst the D_2_O solvent is small enough to enter the cage, the hydrophobic cavity raises the energy of any encapsulated water. Hence, the rate of collision with water molecules is greatly reduced upon encapsulation. The stability of 7 ⊂ 6, can thus be thought of as originating from both a faster rate of formation, and a slower rate of decomposition.

Raymond and co-workers also demonstrated the ability of cage 6 to bind other reactive cations, such as diazonium cation 8 (Fig. 4c).^29^ MOC 6 showed rapid encapsulation of 8 in D_2_O, with hydrophobic and van der Waals interactions between the ethyl groups of 8 and the aromatic walls of cage 6 providing stabilisation. Whilst addition of one equivalent of 8 led to formation of a 1 : 1 host–guest complex, subsequent equivalents of the guest reacted with residual 2,4-pentanedione (left over from the Ga(acac)_3_ used in the cage synthesis). Once encapsulated, cage 6 reduced the reactivity of diazonium 8, through slowing encounters with both the solvent water and 2,4-pentanedione. However, signals from the encapsulated diazonium disappeared after five days, indicating that whilst there was strong binding, guest exchange in and out still occurred, with diazonium 8 becoming more vulnerable during its time free in solution. Encapsulation was substrate specific; diazonium ions 9 and 10 were not encapsulated and thus remained highly reactive. This was attributed to fewer favourable hydrophobic interactions with the cage cavity.

Aromatic tropylium cation 11, which is susceptible to decomposition in water, was also shown to bind in D_2_O. The ^1^H NMR signal for encapsulated tropylium 11 remained sharp after 20 h in solution when protected by MOC 6, whereas it decomposed completely in bulk D_2_O within 24 h.

Raymond and co-workers exploited cage 6 to encapsulate cationic iminium ions in water.^30^ Condensation of amines and ketones to form imines is normally reversible. As water is the by-product, the equilibrium often gets shifted heavily towards the starting materials in water and thus iminiums are only observed as transient species. However, the combination of pyrrolidine and acetone in the presence of cage 6 in aqueous solution gave the inclusion complex 12 ⊂ 6, whereas in absence of the cage no iminium ion 12 was observed (Fig. 4d). The transient iminium formed *in situ* gets readily encapsulated in the hydrophobic cavity, shifting the equilibrium in favour of the iminium. The authors observed this inclusion complex persisted for months, and even when the bulk solution was made basic (which favours iminium hydrolysis). In this report, the size complementarity of guest and host was also demonstrated, with no iminium formation from 2-undecanone, as the corresponding iminium was too large to fit into the cavity.

Raymond and co-workers also showed that tetrahedral MOC 6 promoted the ionisation of organometallic Ru complex 13 to form reactive cationic Ru complex 14 which bound in the cavity (Fig. 4e).^31^ Without cage, cation 14 could be generated from 13 through treatment with AgBF_4_. Cation 14 persisted in CH_2_Cl_2_ for 20 h, but rapidly and irreversibly decomposed in water to form [CpRu(1,3,5-cyclooctatriene)]BF_4_. The authors inferred that 14 was extremely transient in bulk water as no trace was observed outside the cage. However, MOC 6 protected 14 from decomposition when encapsulated and it persisted for weeks in aqueous solution, due to the cage shielding 14 from the water. Although, decomposition was inhibited, inclusion complex 14 ⊂ 6 retained some reactivity. When 14 ⊂ 6 was exposed to (the hydrophobic) CO, it gave a new inclusion complex 15 ⊂ 6. The authors explained that the reaction likely proceeded inside the cage due to the small additional size of CO.

**Fig. 5:**
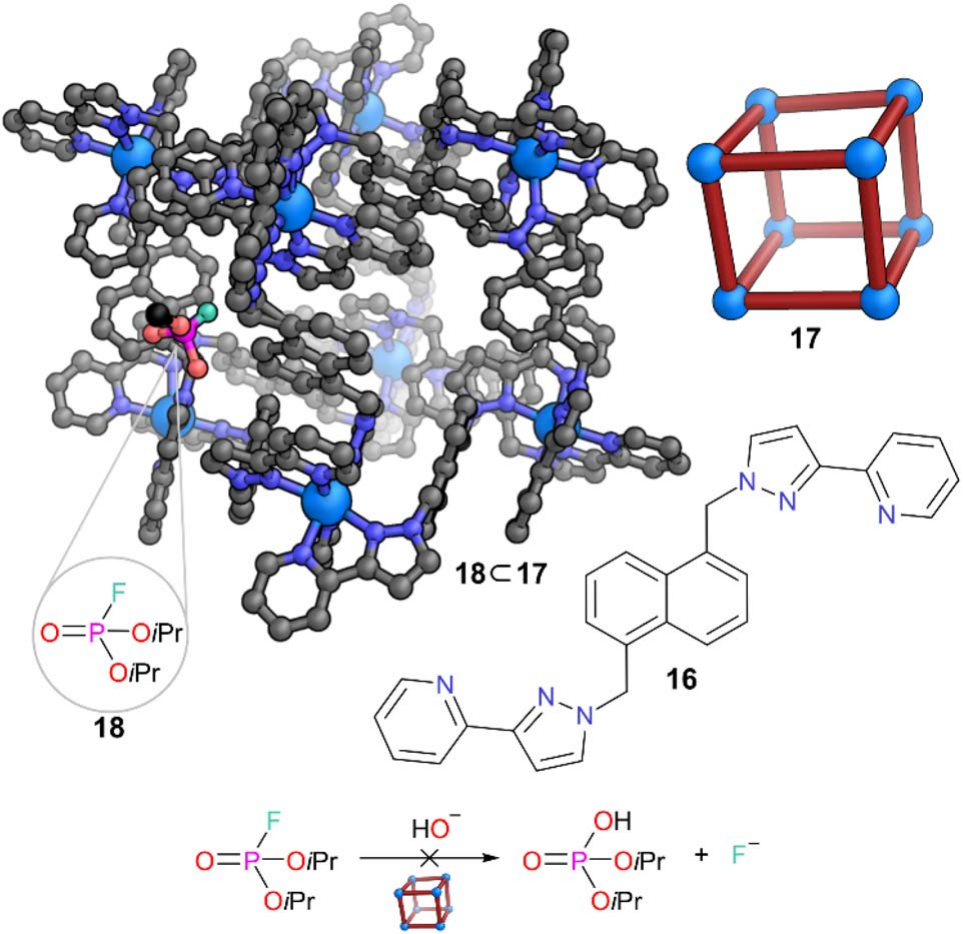
Representation of the SCXRD structure of 18 ⊂ 17. Cage 17 is constructed from ligand 16. A fragment of DFP 18 is shown bound on the face of cage 17 with the P–F bond orientated into the cavity (full structure of DFP was not found due to disorder). Disorder, solvent, hydrogen atoms, and counter anions have been omitted for clarity. Colour: C = grey or black, N = blue, O = red, Co = light blue, P = magenta, F = aquamarine.

In recent years, Ward and co-workers have developed a class of octanuclear cubic coordination cages constructed from bidentate pyrazolylpyridine chelating ligands 16 based on a 1,5-naphthalene-diyl core.^32^ The Co(ii) and Cd(ii) analogues of these cages have shown excellent host–guest properties, binding a range of hydrophobic guests in water. The authors exploited the hydrophobicity of Co(ii) cage 17 to encapsulate and stabilise analogues of chemical warfare agents, such as *O*,*O*′-diisopropyl fluorophosphate (DFP) 18.^33^ These agents are prone to hydrolysis in aqueous conditions at near neutral pH, but interestingly, cage 17 slowed down the hydrolysis significantly through encapsulation (Fig. 5). In buffered solution, the ^19^F resonance corresponding to DFP normally diminished within five days, but when encapsulated in cage 17, this resonance was still observable after 35 days, demonstrating the protection from water MOC 17 provides to the nerve agent analogue. The single crystal structure of host–guest system 18 ⊂ 17 showed that 18 was bound in the window of 17 rather than fully in the cavity. However, the reactive P–F bonds were oriented into the cavity whilst the isopropyl carbon chains were directed outside. Thus, the reactive P centre was placed in a more hydrophobic and sterically hindered environment and was protected from the bulk aqueous medium, explaining the stabilisation obtained from encapsulation.

**Fig. 6:**
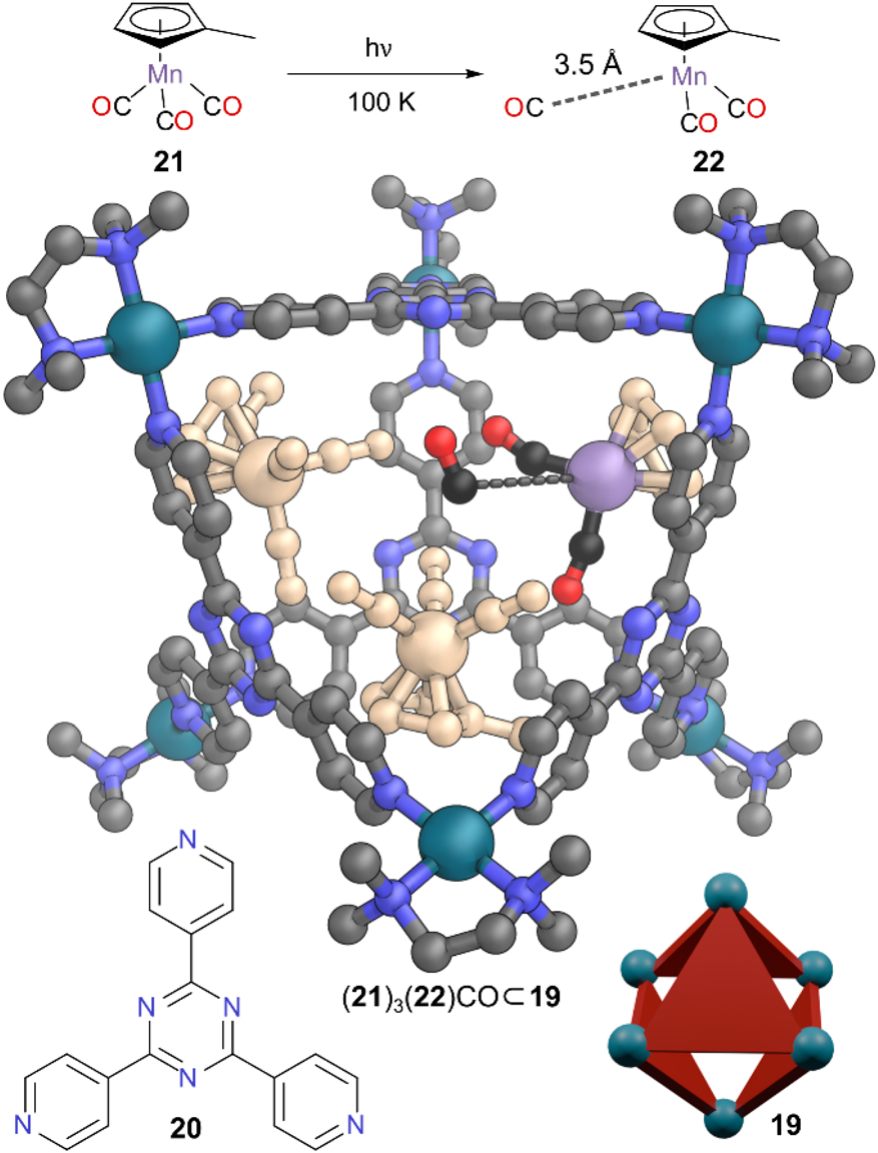
Representation of the SCXRD structure of (21)_3_(22)CO ⊂ 19 constructed from ligand 20. Two Mn tricarbonyls 21 and the one Mn dicarbonyl 22 are shown; the third encapsulated Mn tricarbonyl 21 closest to the camera has been omitted for clarity. Disorder, solvent, hydrogen atoms, and counter anions have been omitted for clarity. Colour: C = grey or black, N = blue, O = red, Pd = turquoise, Mn = purple; other atoms of encapsulated complexes = wheat.

Fujita and co-workers have pioneered preparation of water soluble Pd(ii) MOCs which have shown excellent host–guest properties.^34^ Particularly notable is cage 19, where triangular panels of ligand 20 occupy alternate faces of an octahedral capsule (Fig. 6). Cage 19 has been used extensively to bind neutral guests driven by hydrophobic interactions, with applications in molecular recognition,^35^ separation, and catalysis.^36^ The relatively large hydrophobic cavity in 19, defined by the aromatic walls, promoted the encapsulation of four molecules of organometallic methylcyclopentadienyl manganese tricarbonyl 21, both in aqueous solution and the solid state. When a single crystal of this inclusion complex (21)_4_ ⊂ 19 was irradiated at 100 K, a coordinatively unsaturated manganese complex 22 was observed, providing direct crystallographic evidence of a 16-electron Mn–carbonyl species.^37^ The irradiation promoted dissociation of one CO ligand from one of the four encapsulated Mn complexes. Crystallinity of the sample remained intact and SCXRD data resolved the structure of the 16-electron species, with the dissociated CO trapped in a void more than 3.5 Å away from the unsaturated Mn centre, suppressing chance of recombination. Only one complex showed this CO dissociation, which was attributed to the unavailability of void space in the crystal packing to incorporate more free CO groups. The study of metal carbonyl complexes is an extensive research field,^38^ but information on coordinatively unsaturated species (which are often are key intermediates for organometallic catalytic cycles) remains extremely difficult to determine. This work enabled the geometry of the 16-electron intermediate to be determined as pyramidal. Thus, this is a prime example of how a well-designed cavity can not only stabilise such labile short-lived intermediates but also provide a greater understanding of their structure and reactivity by allowing their observation though experimental techniques not otherwise possible. In solution, they have incredibly short lifetimes as interaction with even alkanes^39^ or xenon^40^ can provide electron density to fill the vacant coordination site. Although this work was done in the solid state, MOCs regularly crystallise with multiple solvent molecules. The combination of the cage structure and the freezing of motion in the solid state were crucial to prevent approach of a replacement donor group from such species.

### Protection from non-aqueous solvent

**Fig. 7:**
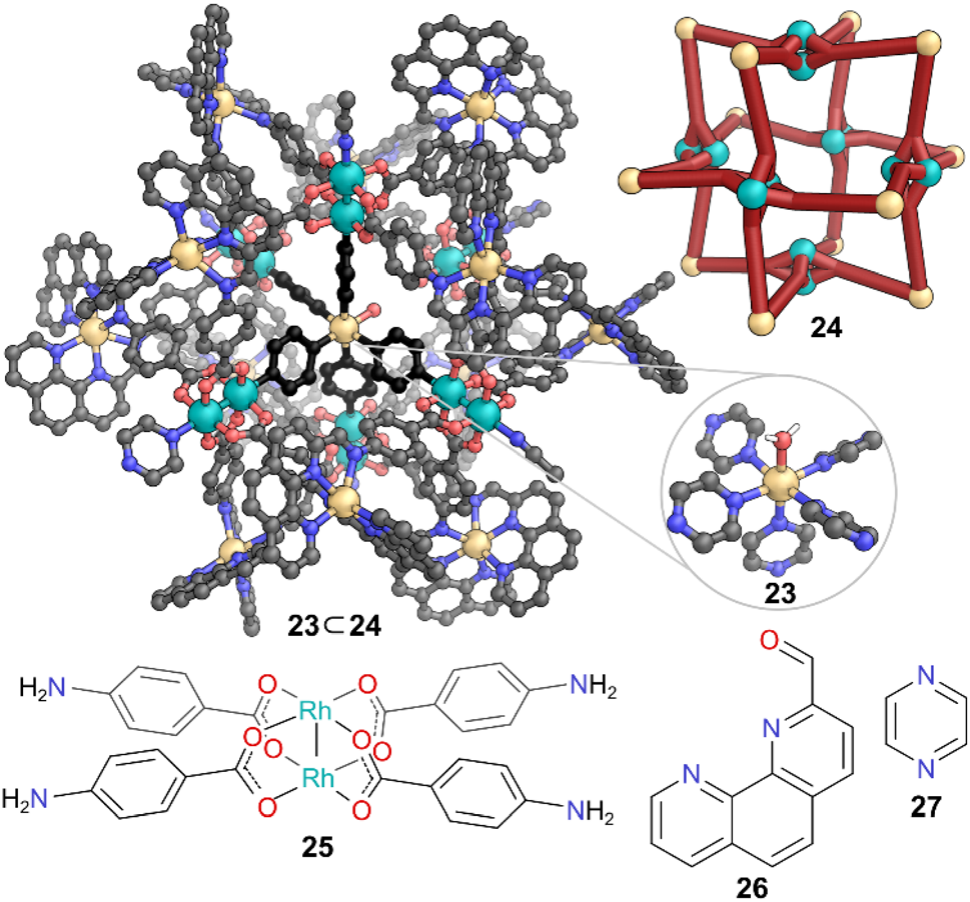
Representation of the SCXRD structure of 23 ⊂ 24. Cage 24 is constructed from components 25 and 26. Disorder, solvent, hydrogen atoms, and counter anions have been omitted for clarity. Colour: C = grey or black, N = blue, O = red, Rh = teal, Cd = light orange.

Nitschke and co-workers demonstrated the formation and stabilisation of pentakispyrazine cadmium(ii) complex 23, which is otherwise energetically unfavourable, by exploiting the confined cavity of cuboctahedral MOC 24.^41^ This cuboctahedron was formed through self-assembly of a dinuclear Rh(ii) paddlewheel 25, 2-formylphenanthroline 26, and Cd(OTf)_2_ (Fig. 7). Rh(ii) dicarboxylate paddlewheels are well-known for exhibiting rich axial coordination chemistry. The authors established the potential binding capabilities of the paddlewheels in 24 through experiments with neutral and coordinating guests. When Cd(OTf)_2_ was added to cage 24 with excess pyrazine 27 an inclusion complex 23 was observed by ^1^H NMR spectroscopy. SCXRD revealed unique cadmium metal complex 23 ⊂ 24, where a Cd(ii) ion, coordinated to five pyrazines and a water molecule, was encapsulated inside the cage cavity, with the other nitrogen of each pyridazine coordinated to the inner Rh of the paddlewheel. To accommodate this guest, a structural distortion from the ideal *O* symmetry of the empty cage was also observed. In previously reported cases, tethering of five pyrazines into a pentadentate ligand was needed to achieve a pentakispyrazine complex. Here, the shape of the confined cavity with available coordination sites and counterions provided the preorganisation to stabilise the Cd(ii) pyrazine coordination complex which is otherwise disfavoured in bulk.

## MOCs preventing reaction with air

**Fig. 8:**
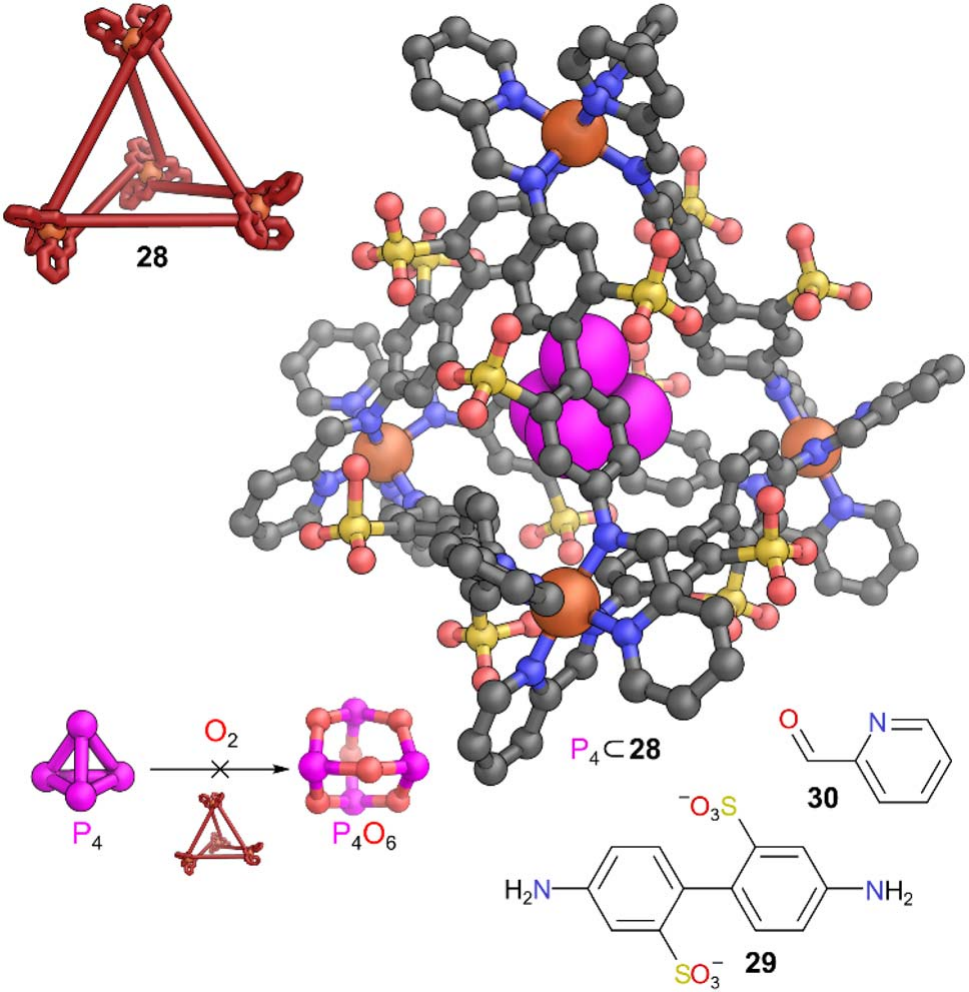
Representation of the SCXRD structure of P_4_ ⊂ 28. Cage 28 is constructed from components 29 and 30. Disorder, solvent, hydrogen atoms, and counter cations have been omitted for clarity. Colour: C = grey, N = blue, O = red, Fe = orange, S = yellow, P = magenta.

Nitschke and co-workers presented one of the landmark examples of stabilising reactive guests inside MOCs by exploiting the tight and hydrophobic microenvironment provided by cage 28 which encapsulated the normally pyrophoric white phosphorus (P_4_) in aqueous solution.^42^ The water-soluble, tetrahedral MOC 28 was constructed from Fe(ii), 4,4′-diaminobiphenyl-2,2′-disulfonic acid 29, and 2-formylpyridine 30. When left in contact with solid white phosphorus, inclusion complex P_4_ ⊂ 28 formed (Fig. 8). This host–guest complex was air-stable for up to four months in aqueous solution. The SCRXD data showed that van der Waals interactions between the P_4_ and the aromatic phenylene groups of the ligands played a pivotal role in overall stabilisation. The tetrahedral geometry of the cage also matched the tetrahedral shape of P_4_, resulting in a favourable tight binding. The key reason for the inertness of P_4_ was not due to oxygen being unable to enter the cage cavity, as an empty cage has enough open space to let O_2_ inside. The size of the cavity was key; P_4_ was already a snug fit. P_4_ reacts with O_2_ to make P_4_O_6_, with an oxygen atom inserting into each P–P bond. This is a multistep reaction with many short-lived intermediates, however, even the addition of a single extra oxygen atom to P_4_ would likely make an intermediate species too large for this cavity. Reaction would therefore require prior expulsion of P_4_ from the cage, a process for which there is a significant energy barrier. Competitive guests such as benzene and cyclohexane displaced the bound P_4_, restoring its reactivity, but molecules too large to be a competitive guest (such as heptane) had no effect. Upon release, the white phosphorus decomposed rapidly, indicating the reactivity of the species was not permanently lost on binding.

**Fig. 9:**
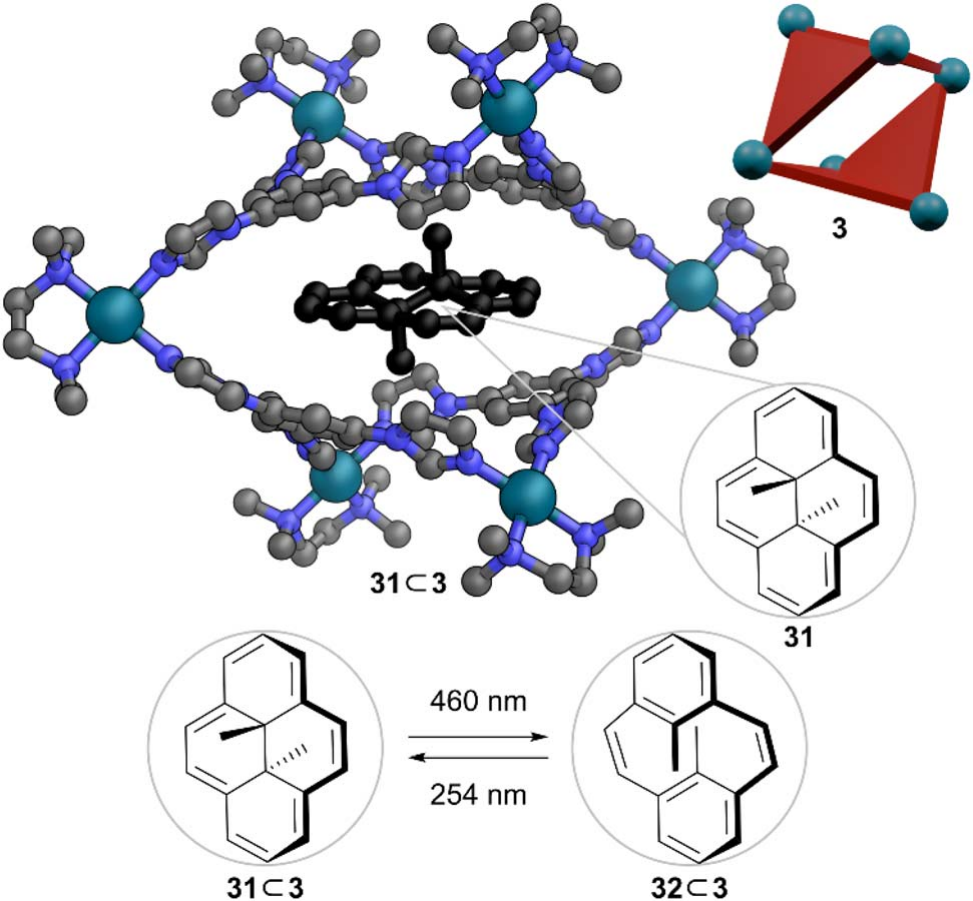
Representation of the SCXRD structure of 31 ⊂ 3 and light promoted interconversion between 31 ⊂ 3 and 32 ⊂ 3. Disorder, solvent, hydrogen atoms, and counter anions have been omitted for clarity. Colour: C = grey, N = blue, Pd = turquoise, DHP = black.

Klajn and co-workers used cavity stabilisation to improve fatigue resistance of dihydropyrene (DHP) switches.^43^ DHP 31 isomerises to cyclophanediene (CPD) 32 upon irradiation with visible light and switches back to DHP with UV light or thermally if kept in the dark (Fig. 9). This process proceeds *via* a diradical intermediate, which is susceptible to decomposition in presence of oxygen. Thus, isomerisation of free DHP in solution leads to fatigue over multiple switching cycles. Previously mentioned, flexible octahedral Pd_6_L_4_ cage 3 developed by Mukherjee and co-workers^6^ was used as the host. A deoxygenated aqueous solution of 31 ⊂ 3 showed only 8% DHP decomposition over 10 cycles to 32 ⊂ 3, compared to 28% decomposition over 10 cycles of free DHP in pentane. The non-deoxygenated experiment showed a 13% decomposition after 10 irradiation cycles, which can be attributed to the easy ingress of oxygen into the cage cavity through the open windows. Nevertheless, a significant improvement in the fatigue resistance of DHP isomerisation in the encapsulated state highlights the ability of MOC 3 to protect the reactive intermediate from oxygen.

**Fig. 10:**
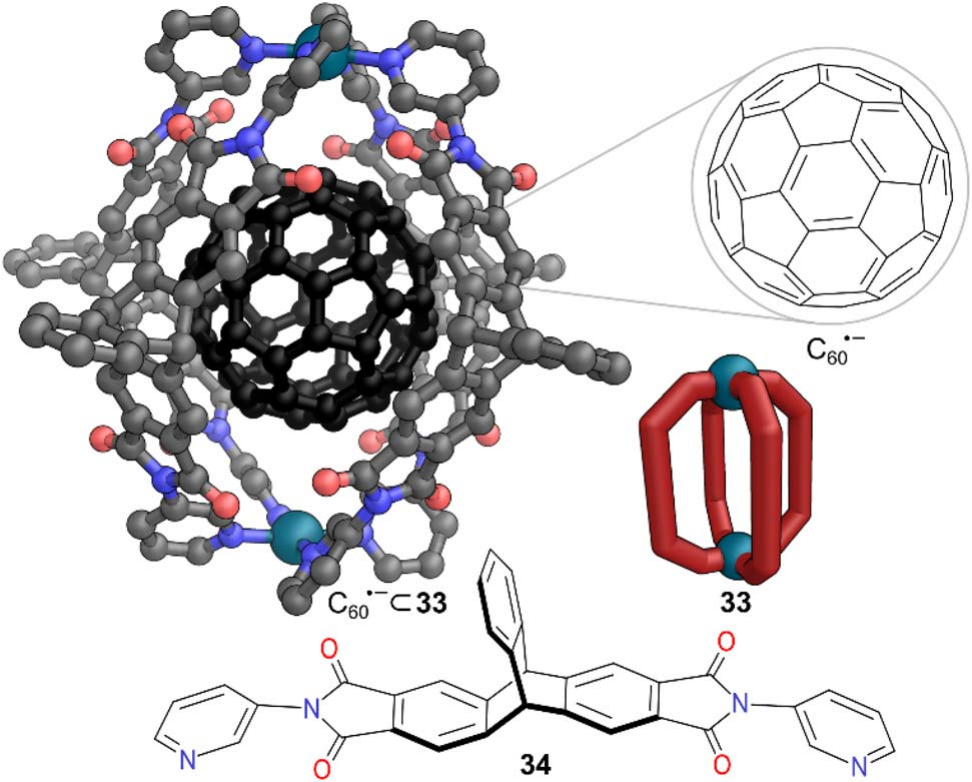
Representation of the structure of C_60_˙^−^ ⊂ 33. Disorder, solvent, hydrogen atoms, and counter anions have been omitted for clarity. Colour: C = grey, N = blue, O = red, Pd = turquoise, C_60_ = black. Note the SCXRD was obtained with C_60_ not the radical anion; the latter is just shown for illustrative purposes.

Clever and co-workers took advantage of cationic Pd(ii) lantern MOC 33, decorated with an electron-deficient, curved inner π-surface, to stabilise C_60_˙^−^, the radical anion formed from one electron reduction of C_60_. C_60_˙^−^ inherently has an extremely short lifetime, making it challenging to investigate with common spectroscopic techniques.^44^ Triptycene-bipyridyl ligand 34 assembled into lantern-shaped MOC 33 with [Pd(CH_3_CN)_4_](BF_4_)_2_. The inner cavity possessed high shape complementarity with the pseudospherical C_60_, allowing easy encapsulation. After encapsulation, C_60_ was photochemically reduced to C_60_˙^−^ (Fig. 10). Owing to the very short lifetime of C_60_˙^−^ direct encapsulation of radical formed in bulk solution was unfavourable. A tight binding was observed due to the cationic nature of the cage and π-surface of the ligands providing stability to the bound guest. The normal lifetime of <1 s was increased to 14 min in aerobic conditions and to ∼300 min in absence of oxygen. EPR signals corresponding to the radical anion were detectable even after a month under inert conditions. Thus, MOC 33 provided stability to the radical anion by kinetically hindering access of oxidants into the cage, leading to the longest reported lifetime of C_60_˙^−^ to date.

## MOCs preventing reaction with other chemicals

**Fig. 11:**
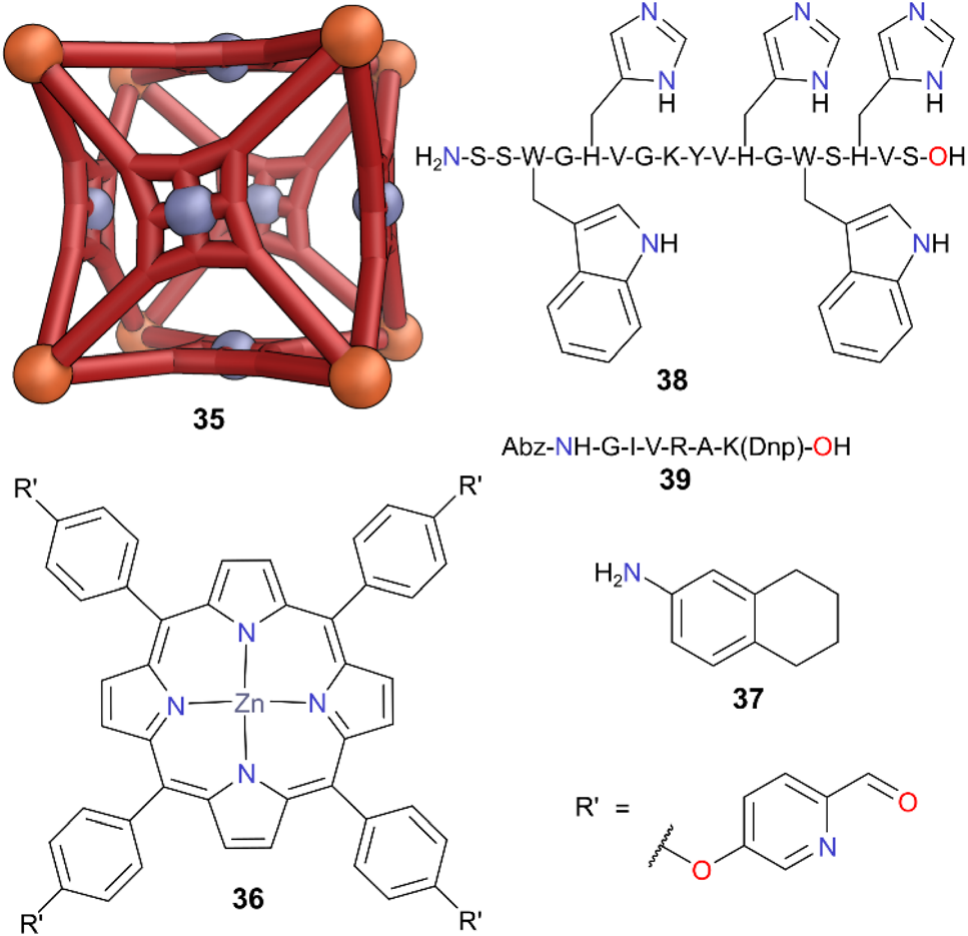
Cartoon representation of cage 35, constructed from components 36 and 37, which binds peptide 38 through the histidines but not peptide 39. Colour: Zn = cool grey, Fe = orange. Peptides are shown by their standard one letter abbreviation for amino acid residues: A = alanine, G = glycine, H = histidine, I = isoleucine, K = lysine, R = arginine, S = serine, V = valine, W = tryptophan, Y = tyrosine. Abz = 2-aminobenzoyl, Dnp = 2,4-dinitrophenyl.

The modular, flexible, and adaptable nature of MOCs is advantageous when it comes to designing synthetic hosts capable of mimicking biological capsules or the functions imparted by compartmentalisation in biology. However, binding complex peptides/proteins is challenging due to their size, flexibility, and reactivity of their amide bonds. This reactivity makes many therapeutic peptides susceptible to enzymatic degradation through amide bond cleavage. Nitschke and co-workers developed large, yet flexible cubic MOC 35 through self-assembly of Fe(ii), square-panelling ligand 36 containing a zinc porphyrin, and tetrahydronaphthylamine 37.^45^ The free axial sites on the zinc(ii) porphyrins internal to this structure enabled cage 35 to encapsulate drug molecules and peptides (Fig. 11). The authors first tested the binding capabilities of cage 35 with small molecule guests able to coordinate to the internal Zn(ii) porphyrin site such as imidazole. Biologically-relevant guests bearing similar motifs (*e.g.* histidine side chains) were then studied in 1 : 1 MeCN : H_2_O solvent and such peptides were encapsulated in cage 35. When peptide 38 (which had two tryptophan chromophores for better recognition *via* HPLC) was treated with the enzyme trypsin in an MeCN/phosphate buffer, it was cleaved in 30 min in 76% yield. The same reaction performed in presence of 0.6 equiv. of cage 35 reduced the cleavage to only 9%. However, when the analogous experiment was performed on non-binding peptide 39 (both with and without cage 35) quantitative enzymatic cleavage was observed. This allowed cage 35 to selectively protect certain reactive species, and it was able to selectively prevent the cleavage of peptide 38 in a mixture of peptides 38 and 39.

**Fig. 12:**
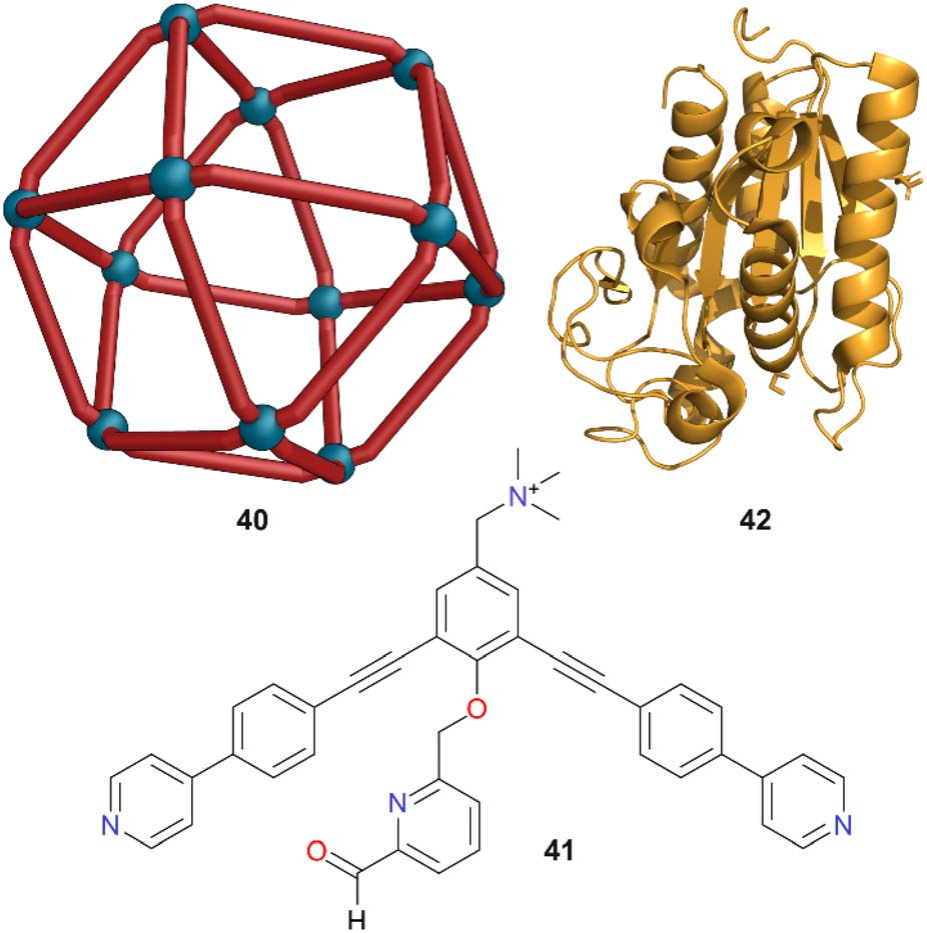
Cartoon representation of cage 40 which is constructed from ligand 41, and CLE enzyme 42. Colour: N = blue, O = red, Pd = turquoise.

Fujita and co-workers reported the synthesis of gigantic Pd_*n*_L_2*n*_ cages by designing ditopic bispyridyl donor ligands with large angles between their coordination vectors.^46^ Pd_12_L_24_ cuboctahedron 40, constructed from ligand 41, has a cavity of ∼5 nm in diameter, and was able to encapsulate individual protein molecules such as the cutinase-like-enzyme (CLE) 42 (Fig. 12).^47^^1^H NMR spectroscopic studies indicated that the folding structure of CLE 42 remained intact after encapsulation. The enzyme's thermal stability was also enhanced, and its degradation was prevented, something which is harder to achieve through encapsulation in micelles or vesicles. An activity assay in a denaturing organic solvent (H_2_O : MeCN (1 : 9) at 20 °C) showed the uncaged CLE had a rapid loss of enzymatic activity (*t*_1/2_ = 1.9 h), but the encapsulated CLE retained high activity even after 6 weeks. Thus, Pd_12_L_24_ cage 40 enhanced the stability of the enzyme 42 by more than three orders of magnitude towards denaturing solvents. Whilst CLE is a relatively stable and compact protein, other proteins with varying hydrophobicity, length, and surface charges could also be encapsulated in other Pd_12_L_24_ cages with different cavity diameters of 4–6 nm.^48^ Through careful matching of size, single protein molecules could be encapsulated selectively. 15 types of proteins were encapsulated, with thermolysin the largest containing 316 amino acids and being 6.4 nm long. These encapsulated proteins retained their enzymatic activity due to the free diffusion possible *via* the large pore openings of the cages. When examined with cytochrome c and proteinase K, the encapsulated proteins showed same activity as their free states.

However, proteins outside the cage in the same organic solvent mixtures were fully deactivated. This enhanced stabilisation against organic solvents whilst retaining activity thus shows great promise for the application of enzymes in more challenging reaction conditions not normally tolerated by biological systems.

**Fig. 13:**
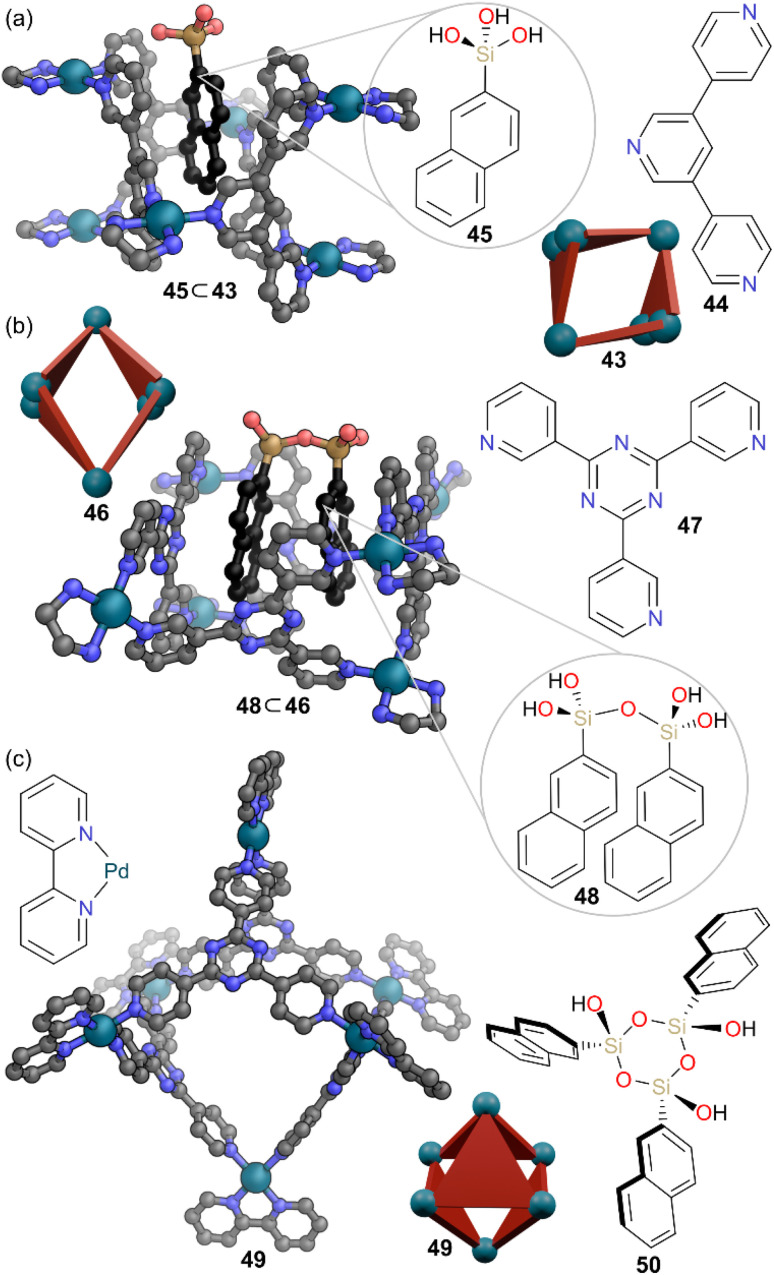
(a) Representation of the SCXRD structure of 45 ⊂ 43, showing the encapsulation of ‘monomeric’ silanetriol 45. (b) Representation of the SCXRD structure of 48 ⊂ 46, showing the encapsulation of silanol dimer 48. (c) Representation of the SCXRD structure of 49 which encapsulates silanol cyclic trimer 50. Disorder, solvent, hydrogen atoms, and counter anions have been omitted for clarity. Colour: C = grey or black, N = blue, O = red, Pd = turquoise, Si = sand.

One of the earliest examples of MOCs protecting reactive species also falls in this class. Fujita and co-workers coined the term “cavity-directed synthesis” to describe the selective oligomerisation of trialkoxysilanes within the cavity of MOCs.^49^ Trialkoxysilanes normally undergo polycondensation which can be used to fabricate silicon materials, however this process can rapidly lead to siloxane networks.^50^ Controlling the degree of oligomerisation is normally difficult, however MOCS were able to regulate the condensation and hydrolysis reactions by encapsulation of intermediates and preventing them reacting with themselves to grow into larger species. Tubular MOC 43, constructed from 3,5-bis(3-pyridyl)-pyridine ligand 44, encapsulated ‘monomeric’ silanetriol 45 within the cavity (Fig. 13a), and despite 45 possessing highly reactive Si–OH groups, reaction with other species was prevented by a tight fit in the cavity. SCXRD indicated strong π–π and C–H–π interactions between the naphthyl group and the cage ligands. Silanetriol 45 was stable for one week within cage 43 in aqueous solution at 80 °C, even in acidic conditions. When the size of the cavity was increased by switching to bowl-shaped MOC 46, constructed from pyridyl triazine ligand 47, selective formation and encapsulation of silanol dimer 48 was observed (Fig. 13b). Further increase again of cavity size to octahedral MOC 49, led to selective encapsulation of the silanol cyclic trimer 50 (Fig. 13c).

**Fig. 14:**
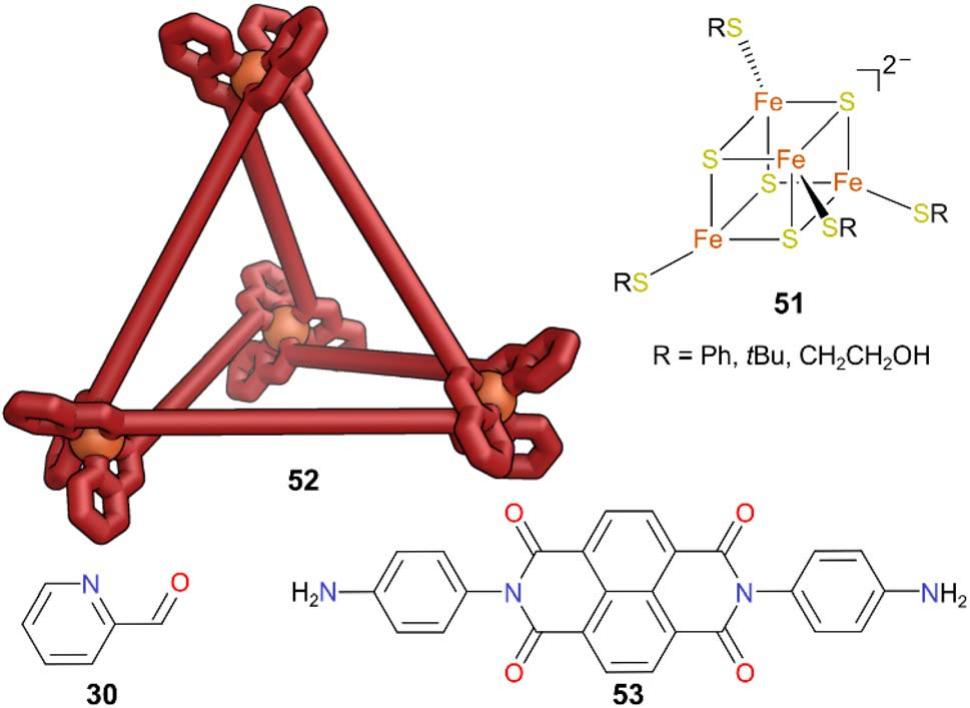
Cartoon representation of cage 52 which is constructed from components 30 and 53. Cage 52 encapsulates cluster 51. Colour: N = blue, O = red, Fe = orange, S = yellow.

A recent report by Bailey and co-workers demonstrated that MOCs can encapsulate and stabilise weak-field synthetic iron-sulfur clusters.^51^ These clusters are particularly important targets for bioinspired synthesis, due to their ability to promote catalytic reactions and serve as model systems to investigate reaction mechanisms. However, due to their instability they are challenging to handle outside of protein scaffolds. In this work, reactive bioinorganic iron–sulfur cofactor 51 was encapsulated in M_4_L_6_ (M = Fe, Ni, Zn) tetrahedral cage 52, constructed from naphthalenediimide (NDI)-containing ligand 53 and 2-formylpyridine 30 (Fig. 14). Dispersion interactions with the NDI panels and electrostatic interactions between the negatively charged cofactor and the positively charged cage promoted cluster encapsulation. The clusters were protected against thermal decomposition by the cage. Under relatively harsh conditions of 120 °C and 3 atm, 44% of cluster remained in the 51 ⊂ 52 sample after 17 days, but only 28% cluster remained in the non-encapsulated control, a nearly twofold improvement. Whilst degradation pathways involving O_2_ or free thiophenol may have been hindered, the ingress of both these species still occurred and excess of these species still caused decomposition.

## MOCs preventing light-promoted reactions

**Fig. 15:**
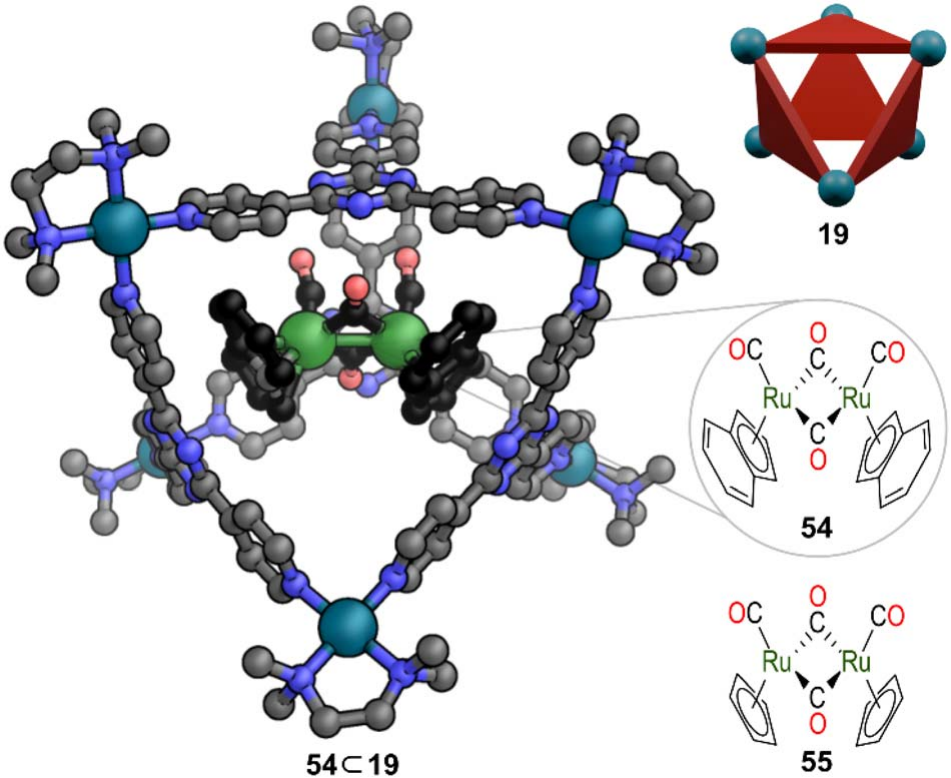
Representation of the SCXRD structure of 54 ⊂ 19. Complex 55 is also encapsulated. Disorder, solvent, hydrogen atoms, and counter anions have been omitted for clarity. Colour: C = grey or black, N = blue, O = red, Pd = turquoise, Ru = dark green.

Fujita and co-workers demonstrated the stabilisation of dinuclear ruthenium complex 54 inside octahedral Pd_6_L_4_ cage 19 in water (Fig. 15).^52^ This Ru complex is normally photosensitive and susceptible to photoinduced Ru–Ru bond cleavage and dissociation of CO. Furthermore, 54 exists as a mixture of *cis* and *trans* isomers, with both bridged and non-bridged forms in equilibrium with each other in solution. Only the *trans* isomer had previously been observed in the solid state.^53^ Interestingly, in this example, MOC 19 encapsulated and stabilised the CO-bridged *cis*-form allowing the first direct SCXRD evidence of the *cis*-form to be obtained. An optimised size fit of 54 into the cavity of 19, along with efficient π–π interactions of the two indenyl groups of 54 with the ligand panels, ensured a tight binding. Hence, even the very small geometry change to interconvert *cis* to *trans* was suppressed within the cage. MOC 19 significantly enhanced the photostability of 54 and no decomposition was observed for several months. When the same encapsulation was examined with less sterically demanding Ru complex 55, again the inclusion complex had the *cis* conformation bound inside. However, the greater amount of free space led to rapid exchange of bridging and terminal CO ligands.

**Fig. 16:**
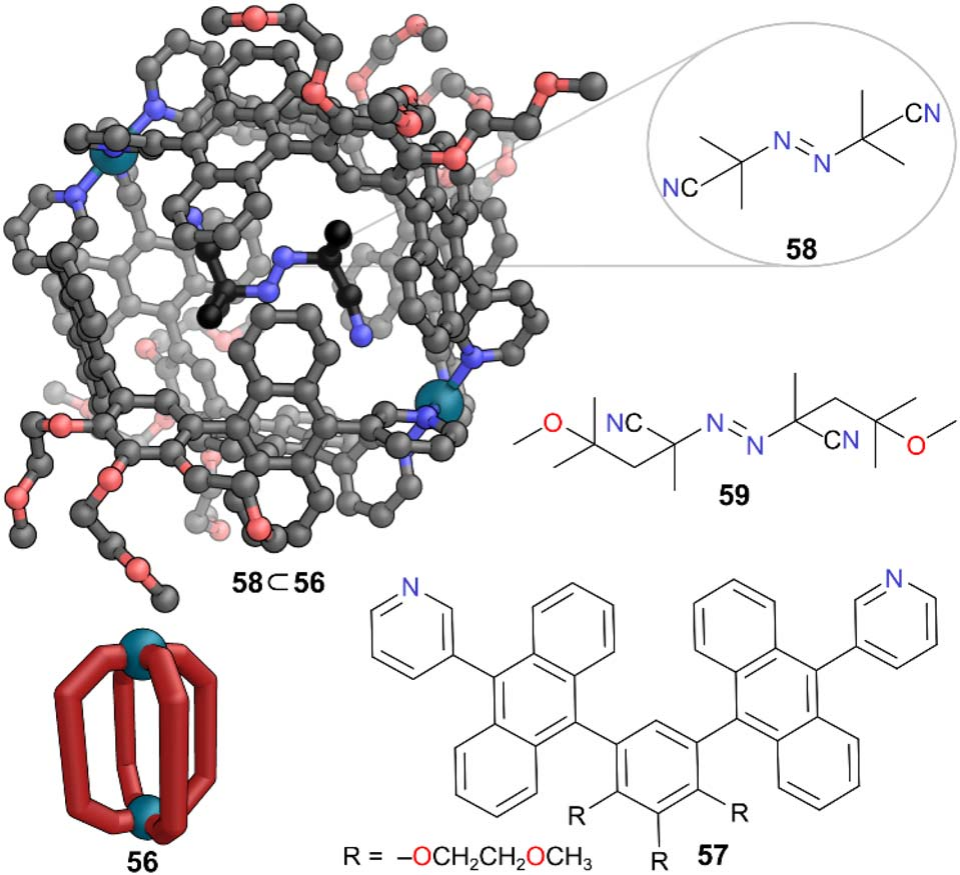
Representation of the SCXRD structure of 58 ⊂ 56. Cage 56 is constructed from ligand 57. Radical initiator 59 is also encapsulated. Disorder, solvent, hydrogen atoms, and counter anions have been omitted for clarity. Colour: C = grey or black, N = blue, O = red, Pd = turquoise.

Another example of a MOC protecting and stabilising reactive species was presented by Yoshizawa and co-workers, where they used a Pd(ii) capsule 56 constructed from ligand 57 to stabilise radical initiators of the azobisisobutyronitrile (AIBN) family.^54^ Radical initiators such as AIBN 58 are unstable to light and heat and must be kept at low temperatures in the dark due to their facile decomposition with the thermodynamically favourable release of N_2_ gas. However, when AIBN 58 was added to a D_2_O : CD_3_CN (9 : 1) solution of MOC 56 at room temperature, formation of 1 : 1 complex 58 ⊂ 56 was observed, driven by the hydrophobicity of the cavity (Fig. 16). SCXRD analysis revealed an S-shaped *trans* conformation for encapsulated AIBN, where the CN groups pointed towards the Pd(ii) centres.

This was further analysed using IR spectroscopy, which suggested weak electrostatic interactions between them also aided the stability of the bound guest. The AIBN initiator remained stable under confinement in the cavity for 10 h under UV irradiation at room temperature, whereas free AIBN decomposes rapidly when free. The half-lives for encapsulated AIBN 58 ⊂ 56 and free AIBN 58 were 690 h and 1.8 h respectively, indicating a 380-fold enhancement in stability to light. The strong absorption of the anthracene-based ligand panels in the visible region occluded the weak absorption of AIBN, thus providing optical shielding. It is also possible that an excited AIBN molecule in the cavity may lose the energy through transfer to the cage rather than through bond cleavage, helping to keep it intact. Other AIBN initiator derivatives encapsulated in cage 56 also showed high stability towards irradiation. Amongst the three radical initiators, the largest one 2,2′-azobis(4-methoxy-2,4-dimethylvaleronitrile) (AMMVN) 59 showed remarkable thermal stability in the bound state, remaining intact for several weeks at room temperature and over 10 h at 50 °C; it decomposed completely in the free state over these times.

**Fig. 17:**
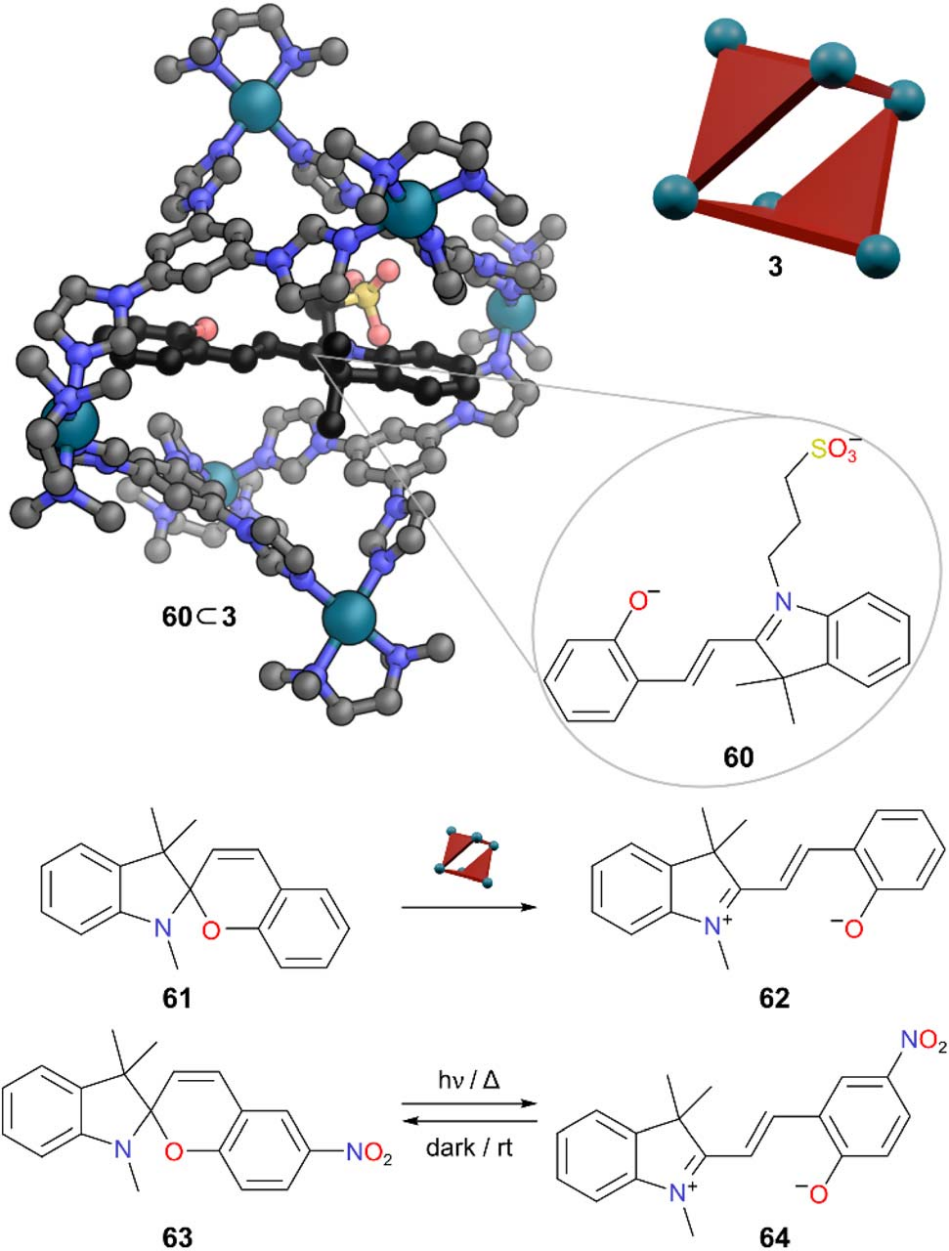
Representation of the SCXRD structure of 60 ⊂ 3. Spiropyran 61 converts to merocyanine 62 which is encapsulated, but merocyanine 64 is not encapsulated. Disorder, solvent, hydrogen atoms, and counter anions have been omitted for clarity. Colour: C = grey, N = blue, O = red, Pd = turquoise, S = yellow.

The following examples of MOCs protecting guests from light involve the stabilisation of “metastable” forms of photochromic molecules. The term metastable typically refers to one isomer (*e.g.*, *cis*) sitting in a potential energy well of higher energy than another isomer (*e.g.*, *trans*). If this metastable isomer can get sufficient energy (typically from a photon of light of appropriate wavelength) it can get over the barrier to isomerise to the lower energy form. Klajn and co-workers used flexible imidazole-based Pd_6_L_4_ cage 3 to encapsulate and stabilise open merocyanine forms of spiropyran derivatives in water which are otherwise unfavoured.^55^ This class of spiropyran photoswitches undergo photoisomerisation between a lower energy ring-closed spiro form and a higher energy (metastable) highly polar open-ring merocyanine form through the cleavage of a C–O bond upon exposure to UV light (Fig. 17). These two forms possess very different properties. The two rings of the spiro form are close to orthogonal, whereas the merocyanine form has a planar zwitterionic structure. This difference in properties governs the nature of encapsulation and stabilisation within the cage cavity. Upon treatment of an aqueous solution of MOC 3 with a spiropyran derivative bearing a sulfonic acid group, encapsulation was observed in the hydrophobic cavity with a spontaneous switching of the spiropyran to the metastable merocyanine form 60. Hydrophobic spiropyran 61 to merocyanine 62 was also switched upon addition of MOC 3, but quantitative 62 ⊂ 3 could not be achieved, likely due to the lower solubility of this spiropyran. Spiropyran 63 normally has the highest proportion of merocyanine form 64 due to the electron withdrawing nitro group enhancing delocalisation in this form. However, merocyanine 64 was not encapsulated in cage 3. This was postulated to be due to the steric clash of the 6′-substituent with the cage wall.

The stability of the bound merocyanine forms was attributed to the tight binding within the cavity with a π–π stacking interaction observed in the SCXRD structure. The release of high-energy water molecules from the cavity of 3 also favoured binding. Irradiation of host–guest complex 60 ⊂ 3 in solution with LEDs near the absorption maximum of the host–guest complex did not show any spectral changes. Conversely, irradiation of free 60 in aqueous solution promoted a ring-closing isomerisation. Thus, the cage was able to invert the relative stability of spiropyran derivatives and stabilise the normally metastable forms through encapsulation.

**Fig. 18:**
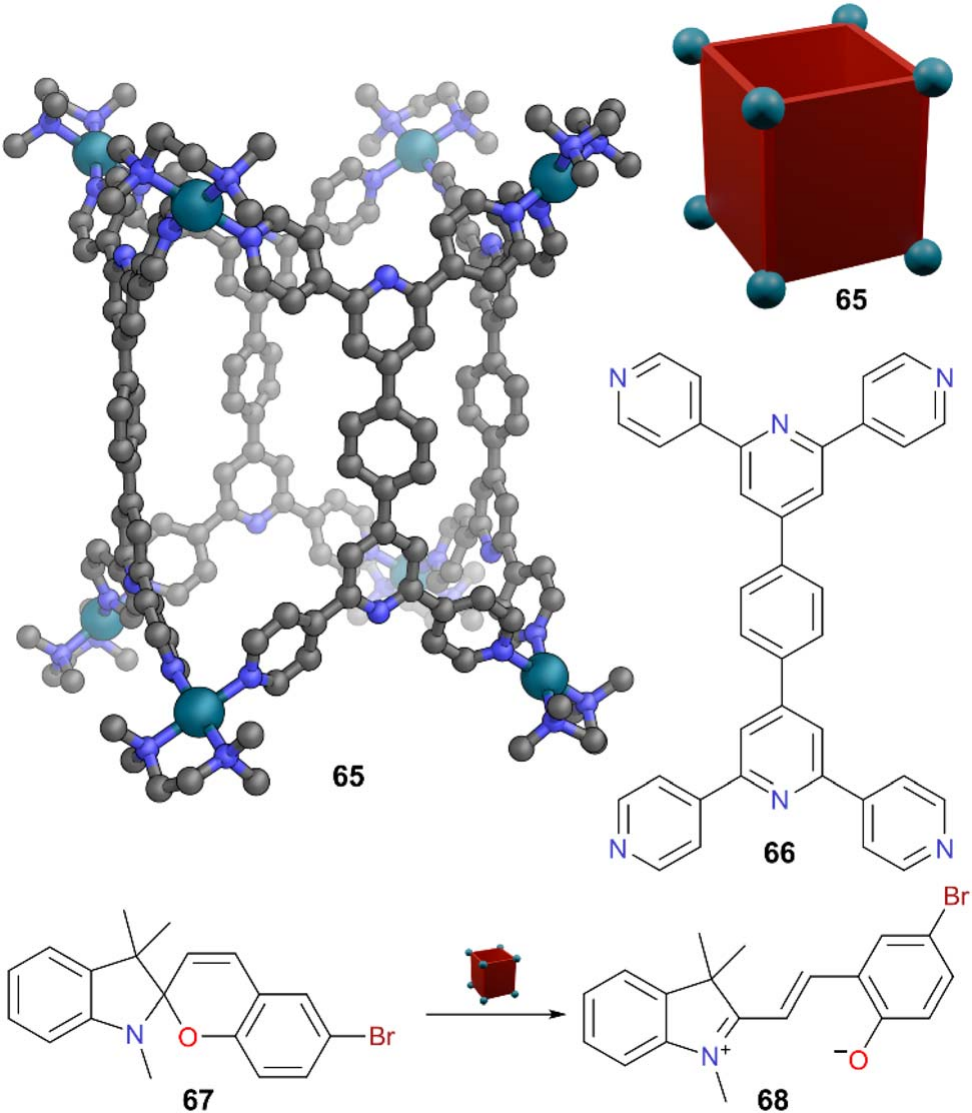
Representation of the SCXRD structure of cage 65 constructed from ligand 64. Spiropyran 67 converts to merocyanine 68 which is encapsulated. Disorder, solvent, hydrogen atoms, and counter anions have been omitted for clarity. Colour: C = grey, N = blue, O = red, Pd = turquoise, Br = brown.

A similar concept was also shown by Mukherjee and co-workers where water soluble molecular barrels were used to encapsulate unstable merocyanine isomers of spiropyran molecules.^56^ Molecular barrel 65 self-assembles from terpyridine-based ligand 66 and *cis*-blocked Pd(ii)-acceptors (Fig. 18). When an aqueous solution of 65 was treated with 6′-bromo spiropyran 67 or 6′-nitro-substituted spiropyran 63, encapsulation of the ring open, planar merocyanine forms 68 and 64 was observed. The larger cavity of 65 allowed the encapsulation of these more sterically demanding guests. These host–guest complexes showed remarkable stability towards visible light, UV light, and heat, demonstrating the strong host–guest interactions provided by the hydrophobic cavity to stabilise the merocyanine derivatives. The authors also generalised the concept, showing similar binding in another water-soluble cage. As a pure substance, spiropyrans are not observed to photoswitch under UV irradiation in the solid state; however, this may be due to facile switching back reducing the lifetime of any merocyanine form. Through grinding, spiropyrans could be encapsulated in these cages in the solid state, and upon irradiation, the cages were able to trap and stabilise the merocyanine form in their hydrophobic pockets, leading to an observable colour change.

Donor–acceptor Stenhouse adducts (DASA),^57^ a new generation of photochromic molecules were reported in 2014. Their photoisomerisation can be promoted by much lower energy visible light and displays contrasting behaviour to spiropyrans. The open conjugated form of DASA is hydrophobic and coloured, and upon irradiation with visible light it photoisomerises to a closed colourless zwitterionic form. In aqueous solution this zwitterionic closed form is stable and it does not isomerise back to the open form.

**Fig. 19:**
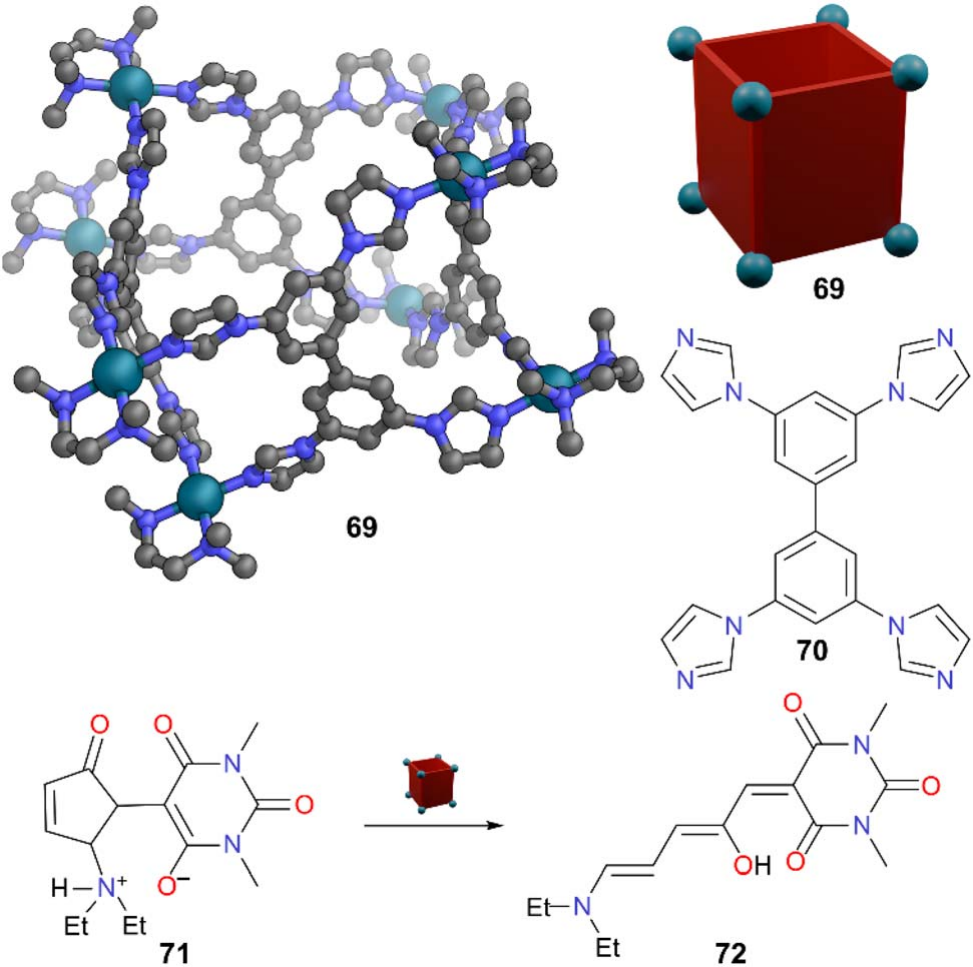
Representation of the SCXRD structure of cage 69 constructed from ligand 70. DASA 71 converts to open form 72 which is encapsulated. Disorder, solvent, hydrogen atoms, and counter anions have been omitted for clarity. Colour: C = grey, N = blue, O = red, Pd = turquoise.

Mukherjee and co-workers investigated the behaviour of DASA molecules inside MOCs.^58^ Water-soluble barrel 69, which is self-assembled from imidazole-based tetratopic donor 70 and a *cis*-blocked Pd(ii) acceptor, encapsulated and stabilised the hydrophobic open form 72 of DASA 71 (Fig. 19). Open form 72 was bound in a 2 : 1 guest : host ratio, with the binding attributed to the hydrophobic microenvironment of the cavity. Given the cyclic form of DASA 71 is most stable in water in the absence of the cage, addition of solid cage 69 can be used to reverse the dominant product at equilibrium to the linear open hydrophobic form by encapsulation. This inclusion complex was stable towards heat and light.

**Fig. 20:**
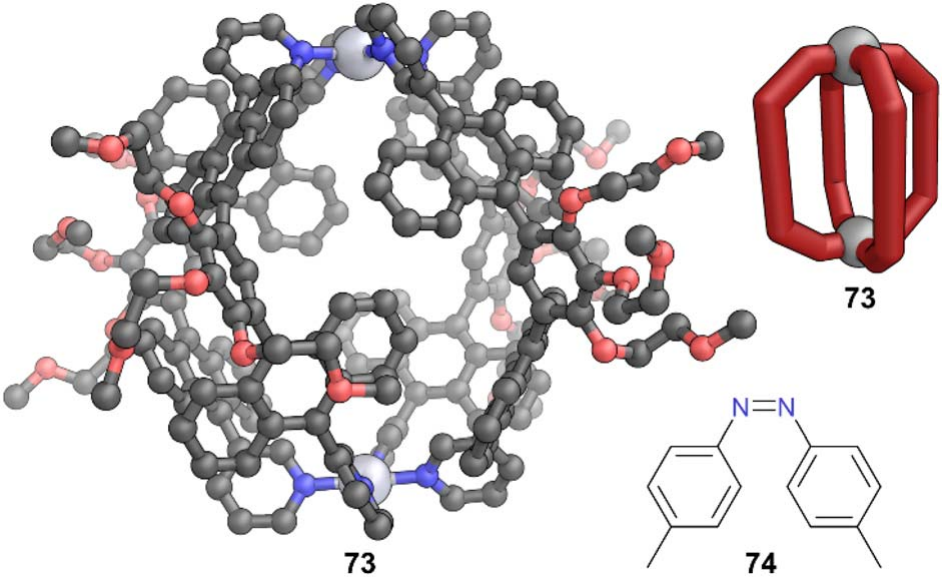
Representation of the SCXRD structure of cage 73, which selectively encapsulates the *cis*-form of azobenzene 74. Disorder, solvent, hydrogen atoms, and counter anions have been omitted for clarity. Colour: C = grey, N = blue, O = red, Pt = light grey.

Azobenzenes are another classic photoswitch; they reversibly isomerise between *cis* and *trans* forms upon irradiation by different wavelengths of light.^59^ Yoshizawa and co-workers examined azobenzenes as guests, and demonstrated the encapsulation and stabilisation of the *cis*-form exclusively in Pt(ii)-based capsule 73, constructed from same ligand 57 as Pd(ii)-based capsule 56.^60^ From a dimethyl substituted *cis*/*trans* mixture of azobenzene 74, MOC 73 selectively encapsulated the *cis* form in aqueous solution in a 1 : 1 host–guest ratio (Fig. 20). The bent *cis*-form was better suited to the spherical cavity where it was stabilised with multiple π–π, C–H–π, and hydrogen bonding interactions, whereas the more linear *trans*-form encountered steric hindrance. The encapsulated *cis*-form did not covert to the *trans*-form upon irradiation with visible light or heating. The authors observed no change in the ^1^H NMR spectrum of the host–guest complex, even after 1 h at 100 °C, which was attributed to the tight binding inside the cavity. By contrast, free *cis*-74 fully converted to *trans*-74 within 20 min at the same temperature. The thermal half-lives calculated for the *cis*–*trans* isomerisation of 74 ⊂ 73 and free 74 were >690 h and 1.4 min respectively. The photoisomerisation under visible light was also slowed by more than five orders of magnitude when encapsulated. More recently, MOC 69 was also shown useful for controlling the rate of conversion between and the relative stability of *E* and *Z*-forms of both stilbene and azobenzene.^61^

Klajn and co-workers also used flexible imidazole-based Pd_6_L_4_ cage 3 to encapsulate monoanionic redox active dyes resorufin and resazurin.^62^ The resazurin/resorufin dyes have been extensively studied in their free state, mostly for biological research. Very different emission properties are observed upon reduction of resazurin to resorufin. Again, cage confinement provided a different microenvironment to bulk solution. Cage 3 provided stability to these guests, and a profound decrease in the kinetics of reduction was observed for resazurin to resorufin and for the further reduction of resorufin to dihydroresorufin.

## MOCs altering functional group reactivity

In this final section we discuss examples of MOCs stabilising a reactive functional group within a molecule containing multiple functionalities. This is typically achieved through partial encapsulation of large molecules. This partial encapsulation enables a normally less reactive functional group which protrudes from the cavity to react in preference to a normally more reactive group buried in the cavity, thereby altering reactivity in the confined cavity compared to bulk solution.

**Fig. 21:**
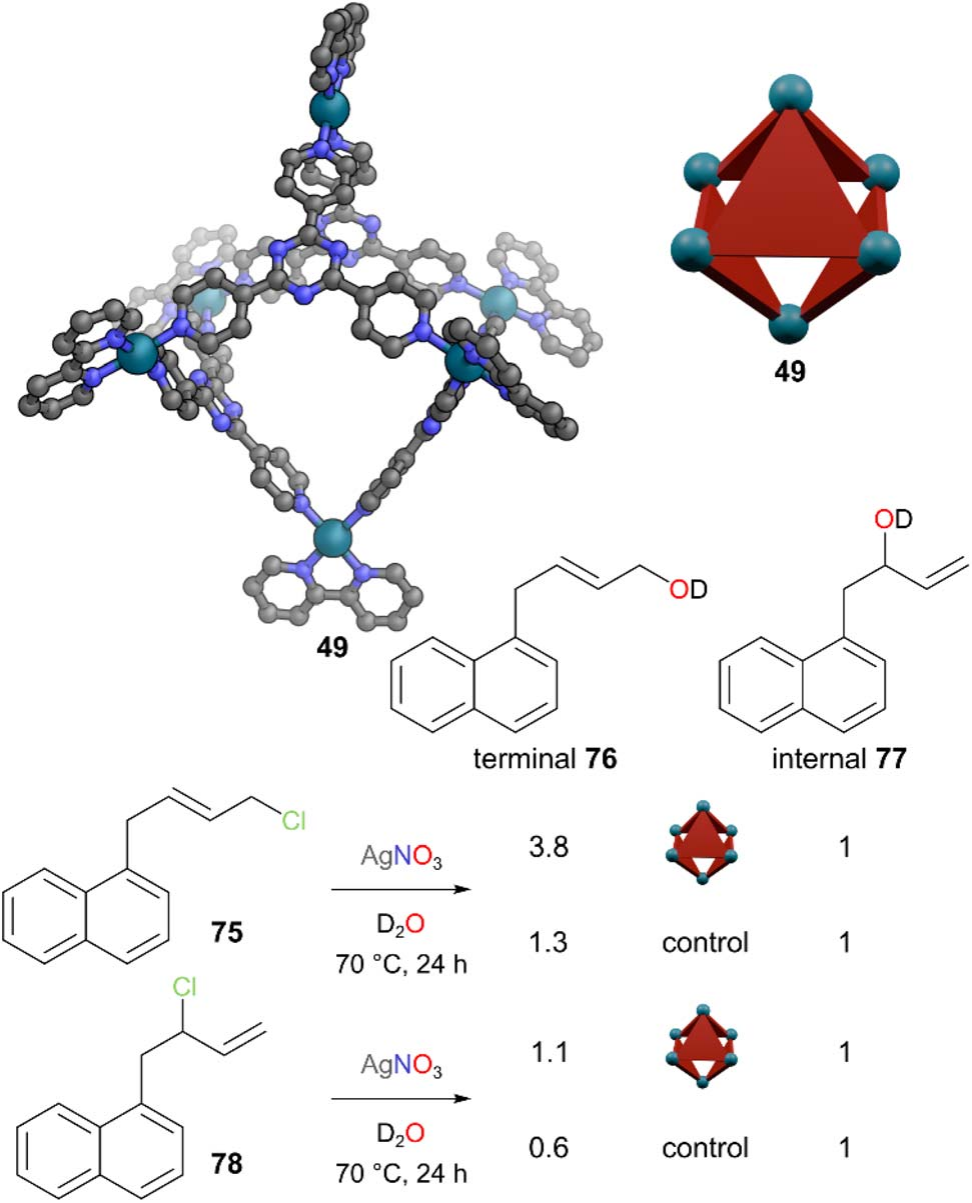
Representation of the SCXRD structure of cage 49. Allylic chlorides 75 and 78 are partially encapsulated within 49, protecting them from nucleophilic attack at the internal position and leading to more of terminal alcohol 76 being formed over internal alcohol 77 than in the absence of cage 49. Disorder, solvent, hydrogen atoms, and counter anions have been omitted for clarity. Colour: C = grey, N = blue, O = red, Pd = turquoise, Cl = light green, Ag = silver.

Nucleophilic substitution of allylic chlorides takes place both at α and γ carbons, leading to two regioisomers.^63^ S_N_1, S_N_2, and S_N_2′ mechanisms are all possible depending on the nucleophile, electrophile, and solvent. For a particular nucleophile/electrophile combination, altering the regioselectivity is difficult and typically limited to changing the solvent polarity. Achieving high selectivity is very difficult. Fujita and co-workers used Pd_6_L_4_ cage 49 to encapsulate aryl-substituted allylic chlorides and shielded the normally more reactive internal reactive site to enhance the regioselectivity for addition of the nucleophile at the terminal position (Fig. 21).^64^ When allylic chloride 75 was encapsulated inside cage 49, there was a significant upfield shift of all signals in the ^1^H NMR spectrum, barring the terminal methylene protons, indicating a greater degree of shielding of the rest of the molecule compared to the terminal reactive site. As attack of the nucleophile at the terminal site was now (relatively) much easier, terminal:internal regioselectivity of 3.8 : 1 was achieved between allylic alcohol products 76 and 77, compared to 1.3 : 1 without cage. Whilst an S_N_1 pathway might be expected in the bulk in the AgNO_3_/H_2_O medium, the differing outcomes of substrates 75 and 78, which would go *via* the same allylic cation in an S_N_1 pathway, indicates at least some tight ion pairing. The regioselectivity of allylic chloride 78 was inverted from 0.6 : 1 terminal : internal in the bulk to 1.1 : 1 inside the cage. Introduction of the competing guest 1-adamantanol supressed the non-covalent protecting group effect of cage 49 as the 1-adamantanol had a higher affinity for the cavity, demonstrating the reversible nature of the non-covalent protection of the γ-carbons by cage 49.

**Fig. 22:**
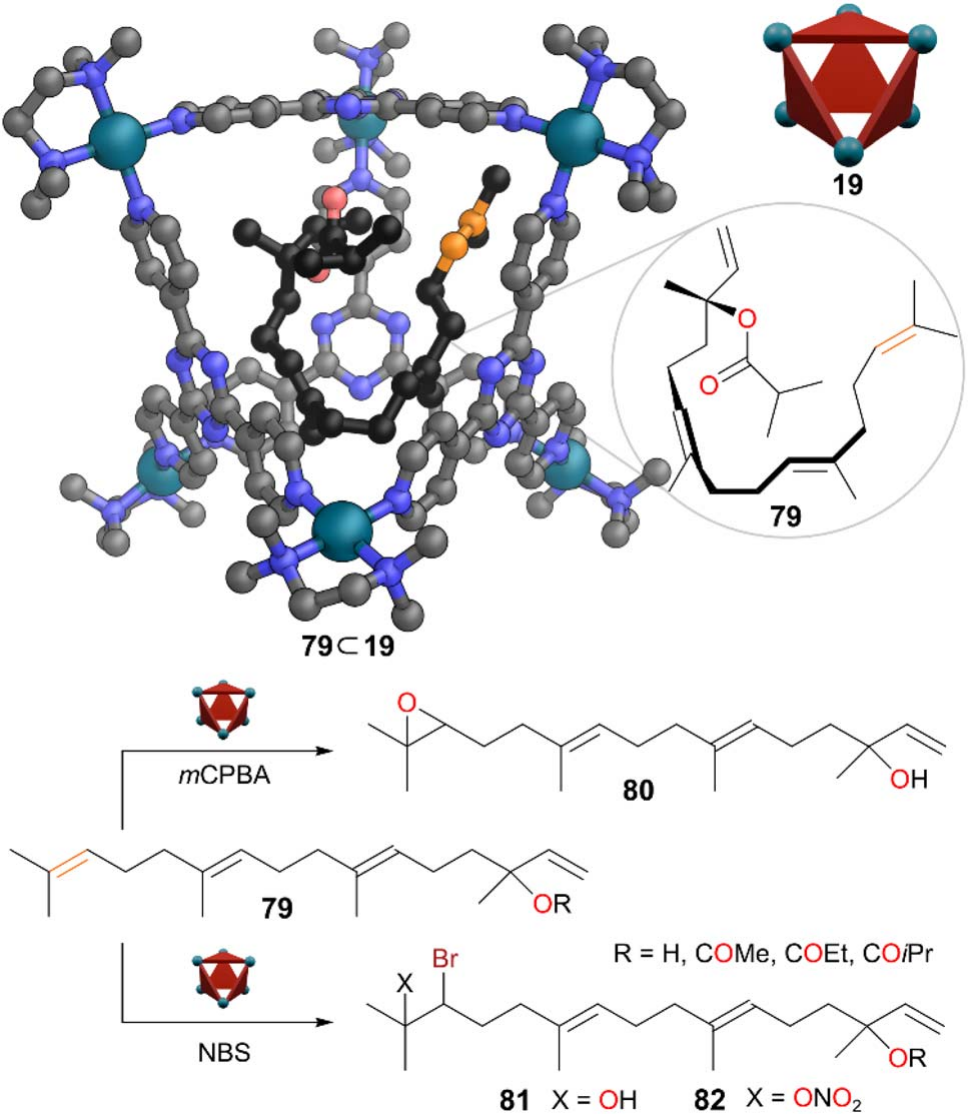
Representation of the SCXRD structure of cage 79 ⊂ 19, and selective reactions of terpene 79 to products 80–82. Disorder, solvent, hydrogen atoms, and counter anions have been omitted for clarity. Colour: C = grey or black, N = blue, O = red, Pd = turquoise, Br = brown, reactive alkene carbons = orange.

Fujita and co-workers also explored encapsulation and reactivity of linear diterpenoids within the cavity of Pd_6_L_4_ cage 19 in water.^65^ Several linear diterpenoids 79 with four C=C bonds (including three trisubstituted C=C bonds of normally similar reactivity) were encapsulated in the cage in a 1 : 1 ratio, with the flexible guests adopting a conformation where the cage could almost fully envelop the diterpenoids (Fig. 22). This observation contrasted with previous studies of linear hydrocarbon binding, where no conformational folding was observed.^66^ From SCXRD analysis, close contact between the two central C=C bonds of 79 and the triazine ligand panel indicated an electrostatic interaction between them. Thus, these central C=C bonds were non-covalently shielded by the cage whereas the third trisubstituted alkene (near the end of the molecule and shown in orange) protruded out of the cavity with less shielding. This observation was supported by the ^1^H NMR spectra of the host–guest assemblies where greater shifts were observed for the protons nearer the centre of the molecule compared to the end. Despite of all the C=C bonds in 79 being susceptible towards electrophiles, only the peripheral trisubstituted alkene reacted. The epoxidation of 79 gave 80 selectively. The bromination of 79 also occurred selectively on the terminal alkene. The bromonium was opened to give mixtures of bromohydrin 81 and the relatively unusual nitratobrominated product 82. Nitratobrominated products are normally difficult to obtain; it likely formed in this case due to the high concentration of nitrate in solution as the cage counter anion. Control experiments on the unbound guests even in the presence of the building blocks of cage 19 showed poor site selectivity with electrophilic substitution occurring on all trisubstituted C=C bonds. Hence, the cage 19 altered the reactivity of these diterpenoids through shielding all but one of the reactive C=C bonds.

**Fig. 23:**
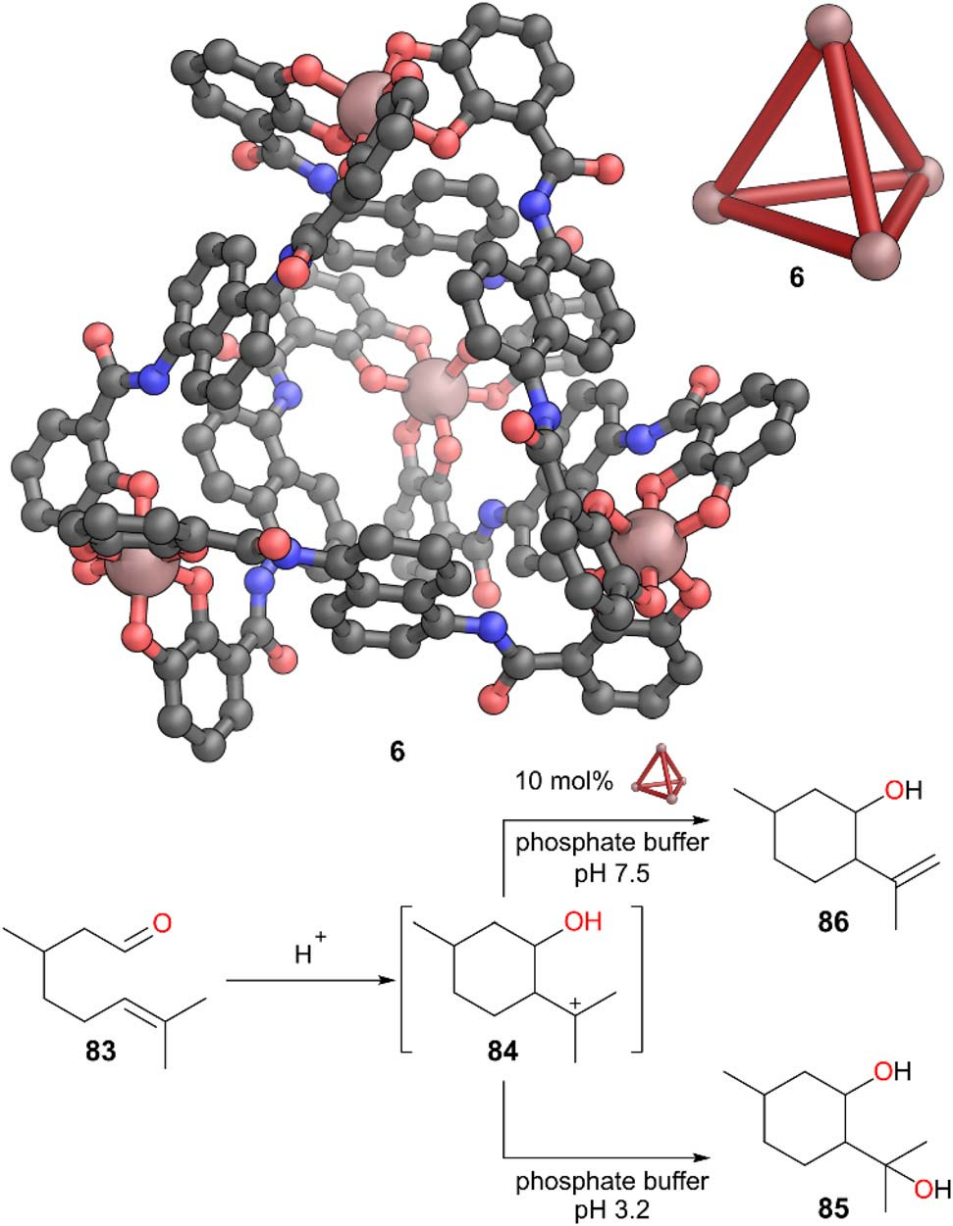
Representation of the SCXRD structure of cage 6 and selective reaction of citronellal 83 to alkene 86 in presence of cage 6. Disorder, solvent, hydrogen atoms, and counter cations have been omitted for clarity. Colour: C = grey, N = blue, O = red, Ga = burnt pink.

Anionic Ga-based cage 6, developed by Raymond and co-workers, has been extensively used for stabilising reactive species as previously discussed. Cage 6 was also able to stabilise the initially formed product in the cyclisation reaction of a monoterpene, inside its hydrophobic cavity (Fig. 23).^67^ Citronellal 83 undergoes Prins-type cyclisation under acidic conditions. The initially formed carbocation 84 is normally trapped by water, leading to large amounts of diol 85 as product. However, cage 6, as well as promoting the formation of this cation, prevents it being attacked by water by providing a hydrophobic cavity. This directs the reactivity of the cation towards a deprotonation pathway to form alkene 86 (Fig. 23). However, upon addition of a slight excess of PEt_4_^+^ as a competing guest, the cyclisation was no longer directed to this product.

## Conclusions

MOCs have numerous advantages, such as their highly tuneable cavities, ease of bespoke design, and adaptable host–guest interactions. This makes them ideal for encapsulating a wide range of substances, ranging in size from single atoms to small proteins. Encapsulation within MOCs offers a robust platform for manipulating and protecting reactive guest species, the control of which is central to chemistry.

Despite the often-destructive nature of reactive species, and the often-unfair categorisation of MOCs as fragile, numerous highly reactive molecules or intermediates have been successfully shielded within MOCs, prolonging their lifetimes and enabling finer control of their reactivity. In this review, we have covered the pioneering early examples through to the most recent exciting discoveries. We categorised examples based on the nature of stabilisation provided by the MOCs, an analysis we believe is needed for more general understanding of this concept to grow. However, this segregation was challenging, as such categorical discussion was often not present in the primary literature, and many MOCs can stabilise a reactive species against multiple deleterious reactivity pathways.

Many early examples involved the cage cavity protecting guests from outside solvents, a feat assisted by the often-hydrophobic nature of the cavities. This was later extended to protection from oxygen in air and other chemicals through encapsulation. Protection of photochromic species from light-promoted reactions, and partial protection of molecules with multiple reactive functionalities has also proved possible. However, this is still a field in its infancy, and if tolerances of a particular MOC to a range of reactivities are fully considered, then there is huge potential.

There are several key concepts we would like the readers to take away from this review and common misconceptions we would like to correct. We hope this will be helpful for the community when it comes to designing strategies to encapsulate reactive guests

(a) Permanent stabilisation of a reactive species is not necessary to have a transformative effect on a process. Enhancement of lifetime is sufficient to allow diversion of reaction pathways, new methods of study to be undertaken, or new applications demonstrated.

(b) The fact that MOCs are not as robust as other classes of porous material should not be seen as a critical limitation; they only need to be unreactive towards the degradation pathway of the reactive species in question. Hence, a specific MOC-guest pairing will often be compatible.

(c) The cavity of an MOC can be easily tailored to a particular guest and their synthesis is often more straightforward and reproducible than other porous material classes, which is important both for reliability and addressing a broad range of challenges.

(d) Complete encapsulation is not required to influence a process. Partial encapsulation is a powerful and underused “non-covalent protecting group” strategy that has advantages of not needing a separate step to both attach and remove the protecting group.

(e) The deleterious reaction partners (water, oxygen, solvent *etc.*) are often smaller than the reactive species in question and can themselves enter the MOC. However, even with this occurring, reactions can still be hugely supressed due to optimised binding of the reactive species in the cavity.

(f) Although MOCs are typically thought of as discrete, solution phase species, the enclosed cavity is very useful in the solid state as well, with the restriction of molecular motion allowing things to be achieved not possible in solution.

(g) Despite substantial advances in MOC design, many of the reported examples here continue to use very well-established Pd(ii) cages from the groups of Fujita and Mukherjee. The continued choice of these systems can be attributed to several factors. They are readily accessible synthetically and have high crystallinity which aids analysis. The choice of capping ligand (*e.g.*, TMEDA) is critical for imparting water solubility and broadening the scope of guest species which can be encapsulated. The cage geometries are well defined, with cavities of precise size and shape; and the presence of some open and some closed faces also aids binding. Pd(ii) centres are advantageous as they show reasonable redox stability, and strike the right balance of coordination bond strength, with the Pd–N bond being sufficiently labile for the reversibility required during synthesis, whilst retaining the strength to give the structures stability under challenging conditions. These would certainly be an ideal starting point for researchers looking to investigate this area.

There are of course some challenges remaining which need attention. The identity of the metal ion influences both the MOC shape and its robustness to certain degradation pathways; sometimes the optimal choice for one of these is not the optimum for the other. Many examples to date also involve rarer/more expensive metal ions, limiting applications that require scalability. Solubility and solvent dependence of binding can also mean properties are not readily transferable from one system to another. However, we believe these challenges will be overcome in time and we hope our review will stimulate readers to find challenging new reactive species to tame and control within metal–organic cages.

## Author contributions

Conceptualisation S. B. and B. S. P.; collecting literature S. B., M. R. B. and B. S. P.; writing – original draft, review and editing S. B., M. R. B. and B. S. P.; visualisation S. B., M. R. B. and B. S. P.; supervision B. S. P.; funding acquisition B. S. P.

## Conflicts of interest

There are no conflicts to declare.

## Data Availability

No primary research results, software or code have been included and no new data were generated or analysed as part of this review.
